# Differential DNA methylation patterns of polycystic ovarian syndrome in whole blood of Chinese women

**DOI:** 10.18632/oncotarget.9327

**Published:** 2016-05-12

**Authors:** Shuxia Li, Dongyi Zhu, Hongmei Duan, Anran Ren, Dorte Glintborg, Marianne Andersen, Vibe Skov, Mads Thomassen, Torben Kruse, Qihua Tan

**Affiliations:** ^1^ Unit of Human Genetics, Department of Clinical Research, University of Southern Denmark, Odense, Denmark; ^2^ Center of Reproductive Medicine, Linyi People's Hospital, Linyi, China; ^3^ Department of Obstetrics and Gynecology, Shandong Medical College, Linyi, China; ^4^ Department of Medicine, Kolding Hospital, Kolding, Denmark; ^5^ Department of Endocrinology, Odense University Hospital, Odense, Denmark; ^6^ Department of Hematology, Roskilde Hospital, Roskilde, Denmark; ^7^ Epidemiology, Biostatistics, and Biodemography, Department of Public Health, University of Southern Denmark, Odense, Denmark

**Keywords:** polycystic ovarian syndrome, DNA methylation, genome-wide association study, clinical heterogeneity

## Abstract

As a universally common endocrinopathy in women of reproductive age, the polycystic ovarian syndrome is characterized by composite clinical phenotypes reflecting the contributions of reproductive impact of ovarian dysfunction and metabolic abnormalities with widely varying symptoms resulting from interference of the genome with the environment through integrative biological mechanisms including epigenetics. We have performed a genome-wide DNA methylation analysis on polycystic ovarian syndrome and identified a substantial number of genomic sites differentially methylated in the whole blood of PCOS patients and healthy controls (52 sites, false discovery rate < 0.05 and corresponding *p* value < 5.68e–06), highly consistently replicating biological pathways extensively implicated in immunity and immunity-related inflammatory disorders (false discovery rate < 0.05) that were reportedly regulated in the DNA methylome from ovarian tissue under PCOS condition. Most importantly, our genome-wide profiling focusing on PCOS patients revealed a large number of DNA methylation sites and their enriched functional pathways significantly associated with diverse clinical features (levels of prolactin, estradiol, progesterone and menstrual cycle) that could serve as novel molecular basis of the clinical heterogeneity observed in PCOS women.

## INTRODUCTION

Polycystic ovarian syndrome (PCOS) is a common endocrinological disorder that affects about 10% women of reproductive age [1–2]. The syndrome has a complex mode of inheritance, in which genomic variants interfere with important environmental factors, including diet, life style, leading to heterogeneous expression of the syndrome characterized by chronic anovulation or infrequent ovulation, obesity, hirsutism, hyperandrogenism and polycystic ovaries. As a complex disorder the pathophysiology of PCOS involves both genetic and environmental contributions [3]. For example, a Dutch twin study [4] estimated a high genetic component in PCOS with a heritability estimate of over 60%. Moreover, previous genetic studies identified multiple genomic loci associated PCOS [5–6]. Meanwhile, animal studies provided evidence that prenatal exposure to excessive androgen induced similar phenotypes to PCOS [7–9] and ovarian dysfunction suggesting the important role of environment in PCOS pathogenesis. The multifactorial nature of PCOS calls for biological functional studies at molecular level to elucidate the integrative mechanisms in the development of PCOS.

Epigenetics focuses on molecular mechanisms in the regulation of gene expression not caused by DNA sequence variation. It represents a new frontier in functional genomics of complex diseases and serves as a potential molecular bridge linking the environment to the genetic materials. Among the various mechanisms of epigenetic regulation, genomic DNA methylation patterns have been widely analysed to investigate the molecular basis of complex disorders mediated by genetic and environmental factors. In the literature, genome-wide association analyses have been performed on PCOS patients and controls by comparing DNA methylation levels between the two groups measured using high-throughput techniques [10–13]. However, results from the different genome-wide analyses have been highly inconsistent. For example, while Xu et al. [10] reported no significant difference in the DNA methylome of peripheral blood cells of 20 PCOS patients and 20 controls, multiple differentially methylated genes were identified by Shen et al. [11] in peripheral blood from even a smaller sample size. By targeting the ovarian tissue, large numbers of differentially methylated genes were found by both Wang et al. [12] and Yu et al. [13] in their small studies. Nonetheless, the detected genes from each study were associated with different molecular functions even though both studies were conducted on the ovarian tissues. The situation calls for well-designed studies on relatively large sample sizes to validate and update current findings to look for novel genomic sites and biological pathways associated with PCOS.

This paper reports our recent epigenome-wide association study (EWAS) on a relatively large sample size of 30 PCOS patients and 30 age-matched healthy controls. We present results from analysis on single CpG sites (5′—C—phosphate—G—3′, cytosine and guanine separated by one phosphate) followed by findings on enriched biological pathways significantly associated with PCOS condition. Furthermore, we report our novel analysis in associating genomic DNA methylation with levels of reproductive hormones in PCOS patients including estradiol (E2), luteinising hormone (LH), follicle stimulating hormone (FSH), progesterone (P), thyroid stimulating hormone (TSH), prolactin (PRL), testosterone (TST), which could reveal the molecular basis of the observed clinical heterogeneity in PCOS patients.

## RESULTS

Table 1 presents the basic statistics for both PCOS and control samples on phenotypes of interest including anthropometric measurements: weight, height, body mass index (BMI), waist and hip circumference, waist-hip-ratio (WHR); blood pressure: systolic and diastolic blood pressure (SBP, DBP); menstrual cycle (MC); reproductive hormones: E2, LH, FSH, P, TSH, PRL and TST; and metabolic variables: fasting immunoreactive insulin (IRI), immunoreactive insulin at 2 hours after ingestation of 75 gram dextrose (IRI2), fasting blood glucose (GLU), blood glucose at 2 hours after ingestation of 75 gram dextrose (GLU2), homeostatic model assessment of insulin resistance (HOMA-IR). Highly significant differences were found for MC, LH and TST between the two groups; statistical or borderline differences were also found for WHR (*p* = 0.037), IRI2 (*p* = 0.026) and GLU2 (*p* = 0.031), all with higher levels in PCOS patients than in controls.

**Table 1:** Descriptive statistics of PCOS and control samples

	PCOS, *n* = 30			Control, *n* = 30			
	Median	2.5%	97.5%	Median	2.5%	97.5%	*P* value
Age, year	25	23	30	27	24	31	
Weight, kg	61	47.5	116.9	63	47.8	86	0.90
Height, cm	160	149.8	175.3	160	154.3	168.3	0.19
BMI, km/m2	23	19.4	38	23.4	18.1	33.6	0.67
Waist, cm	80	56.1	114.8	81	66.3	101.5	0.75
Hip, cm	97	84.8	123.3	98	88.8	115	0.68
WHR, %	83.7	75.7	102.8	81.1	72.7	94.5	0.04
SBP, mmHg	110	100	130	110	90	132.5	0.95
DBP, mmHg	70	60	83.8	70	66	90	0.07
MC, day	75	28	407	30	25	35	7.09e–10
E2, pg/ml	49.9	13.8	151.5	45.5	27.9	115.9	0.38
LH, mIU/ml	14.1	3	25	4.4	1.6	12	5.12e–08
FSH, mIU/ml	6.4	4.8	8.9	6.3	3.4	11.1	0.63
P, ng/ml	0.4	0.2	1.1	0.4	0.1	0.9	0.99
TSH, uIU/ml	1.8	0.5	5.4	1.7	0.6	4.7	0.79
PRL, ng/ml	10.4	4	55	11.3	5.8	27.2	0.97
TST, ng/dl	50.6	6.2	94.7	31.4	7.6	62.8	3.30e–04
IRI, uIU/ml	15.3	2.1	51.6	10.5	5.6	27.3	0.11
IRI2, uIU/ml	66.4	10.4	300	46.5	13.7	196.5	0.03
GLU, mmol/l	5.2	4.8	5.7	5.3	4.8	5.9	0.31
GLU2, mmol/l	6.7	4.5	8.8	6.0	3.5	8.4	0.03
HOMA-IR	3.5	0.5	12.4	2.3	1.3	6.2	0.15

Abbreviations: WHR: waist to hip ratio; SBP: systolic blood pressure; DBP: diastolic blood pressure; MC: menstrual cycle; E2: estradiol; LH: luteinising hormone; FSH: follicle stimulating hormone; P: progesterone; TSH: thyroid stimulating hormone; PRL: Prolactin; TST: Testosterone; IRI: immunoreactive insulin; IRI2: IRI at 2 hours after ingestion of 75 g dextrose; GLU: glucose; GLU2: GLU at 2 hours after ingestion of 75 g dextrose; HOMA-IR: homeostatic model assessment of insulin resistance.

### Epigenetic association with PCOS

**Figure 1:**
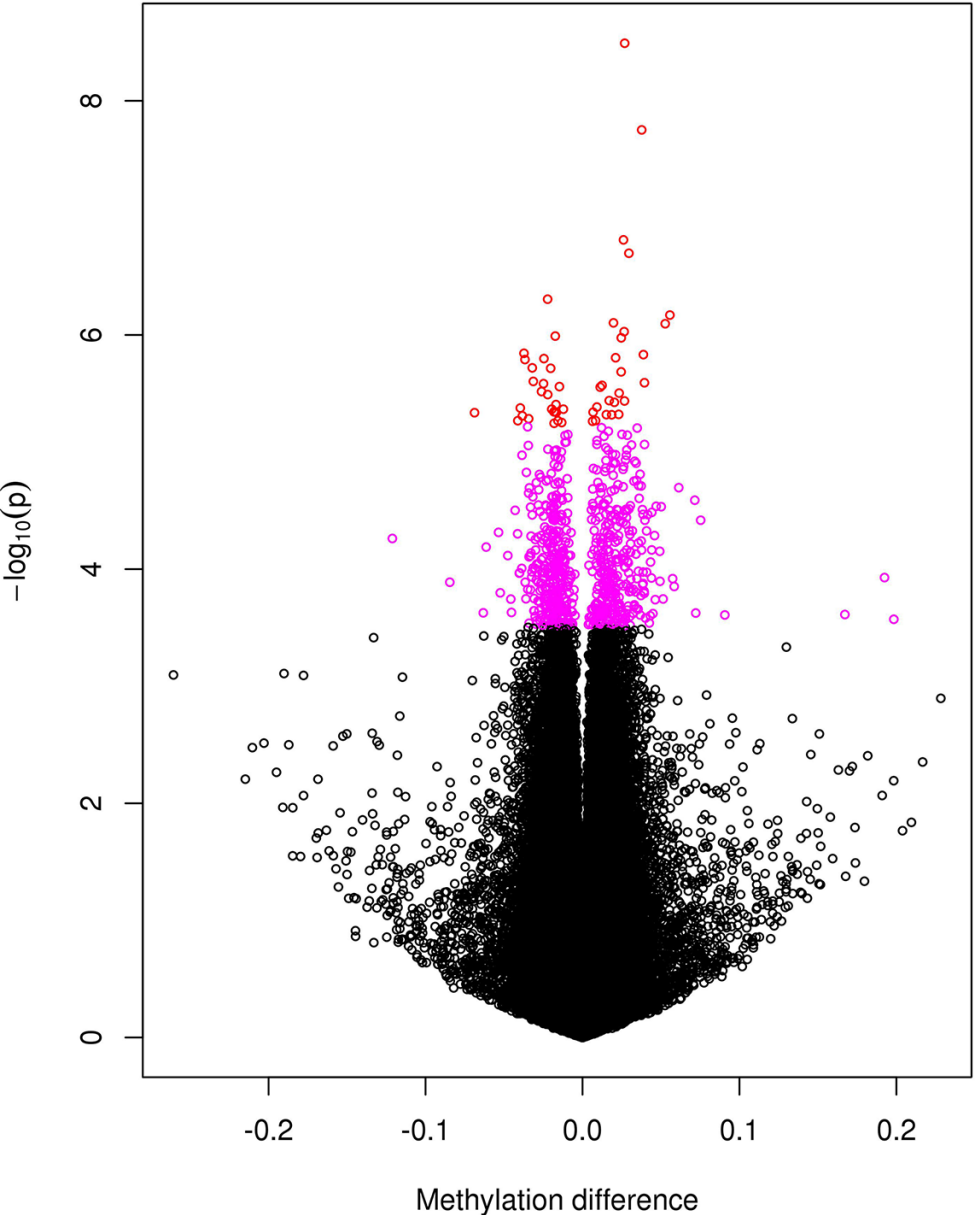
A volcano plot for the negative log10-transformed *p* values plotted against the difference in the mean levels of DNA methylation at each CpG site between PCOS and controls groups CpGs reaching genome-wide significance with FDR < 0.05 are coloured red and those with 0.05 < FDR < 0.2 coloured purple.

We first performed EWAS for single CpGs (Manhattan plot shown in Supplementary Figure S1A). After correction for multiple testing, a total of 699 CpGs (13 X-linked) were found with false discovery rate (FDR) < 0.20 with corresponding *p* value < 3.05e-04 (Supplementary Table S1), among them 52 CpGs (1 X-linked) with FDR < 0.05 with corresponding *p* value < 5.68e-06. As shown in Supplementary Table S1, the mean methylation levels of significant CpGs range from low to high but are dominated by sites of high DNA methylation levels. Figure 1 is a volcano plot displaying *p* value (in log scale) plotted against corresponding difference in the mean methylation levels between PCOS patients and controls. The coloured spots represent 699 CpGs with FDR < 0.2, among them the red spots stand for the 52 genome-wide significant CpGs with FDR < 0.05 in Supplementary Table S1. The figure displays the significance level for hyper- and hypo-methylated CpGs without a predominant pattern of increased or decreased methylation in patient or control group. The figure also shows a symmetric pattern although the top significant CpGs tend to be hypermethylated (i.e. increased in mean methylation level) in the patient group. Both Supplementary Table S1 and Figure 1 show that the significant CpGs are those with only small differences in their DNA methylation levels between the two groups.

Based on the EWAS results, we conducted a gene-set enrichment analysis (GSEA, see Methods section) on the 273 genes linked to the 699 significant CpGs in Supplementary Table S1. A total of 22 functional pathways were significantly enriched with FDR < 0.05 (Table 2). Among the 22 pathways, the top significant ones are mainly those involved in immune and inflammatory processes with the rest pertaining to biological processes including metabolism of proteins and carbohydrates. There are in total 4 pathways with FDR < 0.01(the very top of Table 2), all are involved in immunity (intestinal immune network for IgA production; asthma; O-Glycan biosynthesis) and inflammation (viral myocarditis).

**Table 2:** The 22 functional pathways enriched (FDR < 0.05) by genes linked to CpG sites in Supplementary Table S1

Gene Set Name [# Genes (K)]	Description	# Genes in Overlap (k)	*p*-value	FDR (*q*-value)
KEGG: VIRAL MYOCARDITIS [73]	Viral myocarditis	6	5.07 e^−6^	6.59 e^−3^
KEGG: INTESTINAL IMMUNE NETWORK FOR IGA PRODUCTION [48]	Intestinal immune network for IgA production	5	9.91 e^−6^	6.59 e^−3^
KEGG: ASTHMA [30]	Asthma	4	2.96 e^−5^	9.83 e^−3^
KEGG: O–GLYCAN BIOSYNTHESIS [30]	O–Glycan biosynthesis	4	2.96 e^−5^	9.83 e^−3^
KEGG: LEISHMANIA INFECTION [72]	Leishmania infection	5	7.21 e^−5^	1.46 e^−2^
REACTOME: METABOLISM OF PROTEINS [518]	Genes involved in Metabolism of proteins	12	7.33 e^−5^	1.46 e^−2^
KEGG: ALLOGRAFT REJECTION [38]	Allograft rejection	4	7.67 e^−5^	1.46 e^−2^
REACTOME: ADAPTIVE IMMUNE SYSTEM [539]	Genes involved in Adaptive Immune System	12	1.06 e^−4^	1.69 e^−2^
KEGG: GRAFT VERSUS HOST DISEASE [42]	Graft–versus–host disease	4	1.14 e^−4^	1.69 e^−2^
KEGG: TYPE I DIABETES MELLITUS [44]	Type I diabetes mellitus	4	1.37 e^−4^	1.82 e^−2^
REACTOME: POST CHAPERONIN TUBULIN FOLDING PATHWAY [19]	Genes involved in Post–chaperonin tubulin folding pathway	3	1.87 e^−4^	2.22 e^−2^
KEGG: SYSTEMIC LUPUS ERYTHEMATOSUS [140]	Systemic lupus erythematosus	6	2.01 e^−4^	2.22 e^−2^
REACTOME: HEPARAN SULFATE HEPARIN HS GAG METABOLISM [52]	Genes involved in Heparan sulfate/heparin (HS–GAG) metabolism	4	2.64 e^−4^	2.54 e^−2^
KEGG: AUTOIMMUNE THYROID DISEASE [53]	Autoimmune thyroid disease	4	2.84 e^−4^	2.54 e^−2^
REACTOME: ANTIGEN PROCESSING UBIQUITINATION PROTEASOME DEGRADATION [212]	Genes involved in Antigen processing: Ubiquitination & Proteasome degradation	7	2.97 e^−4^	2.54 e^−2^
KEGG: INOSITOL PHOSPHATE METABOLISM [54]	Inositol phosphate metabolism	4	3.05 e^−4^	2.54 e^−2^
REACTOME: O–LINKED GLYCOSYLATION OF MUCINS [59]	Genes involved in O–linked glycosylation of mucins	4	4.29 e^−4^	3.36 e^−2^
PID AR PATHWAY [61]	Coregulation of Androgen receptor activity	4	4.87 e^−4^	3.6 e^−2^
REACTOME: IMMUNE SYSTEM [933]	Genes involved in Immune System	15	5.26 e^−4^	3.68 e^−2^
REACTOME: METABOLISM OF CARBOHYDRATES [247]	Genes involved in Metabolism of carbohydrates	7	7.37 e^−4^	4.9 e^−2^
REACTOME: CLASS I MHC MEDIATED ANTIGEN PROCESSING PRESENTATION [251]	Genes involved in Class I MHC mediated antigen processing & presentation	7	8.1 e^−4^	4.98 e^−2^
REACTOME: HS GAG BIOSYNTHESIS [31]	Genes involved in HS–GAG biosynthesis	3	8.24 e^−4^	4.98 e^−2^

### Epigenetic association with clinical features in PCOS patients

**Figure 2:**
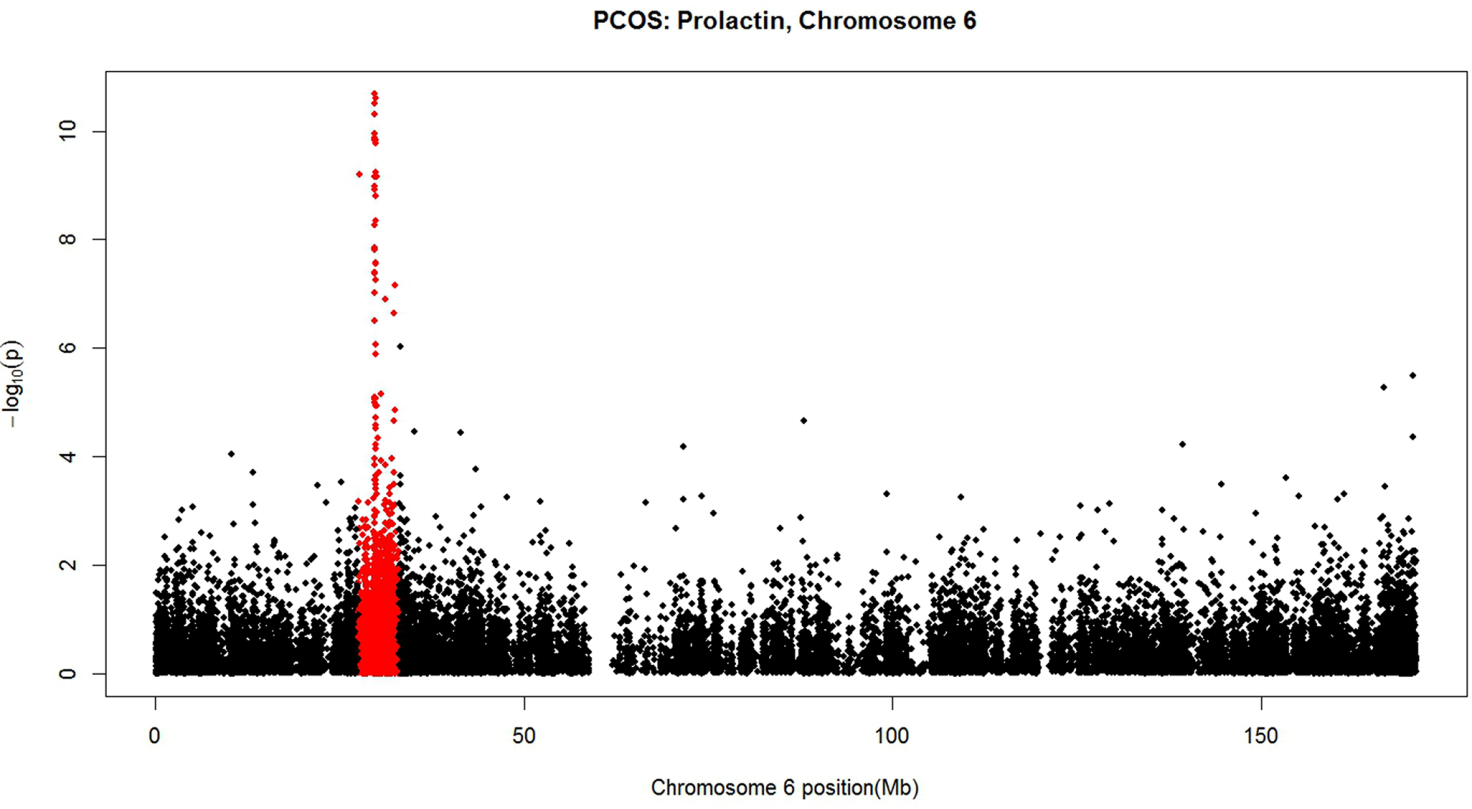
A Manhattan plot for the negative log10-transformed *p* values for PRL in PCOS patients plotted against chromosome location (Mb) for each CpG alone chromosome 6 The HLA region highlighted with red colour harbours CpGs highly associated with PRL.

In addition to comparing DNA methylation between PCOS patients and controls, we also conducted EWAS on the 30 PCOS patients for their clinical features including BMI, MC, reproductive hormones (E2, LH, FSH, P, TSH, PRL, TST), and metabolic variables (IRI, IRI2, GLU, GLU2, HOMA-IR). Multiple CpGs reaching genome-wide significance (FDR < 0.05) were found for E2 (87 CpG sites, corresponding *p* value < 8.36e-06, Supplementary Table S2); for PRL (199 CpG sites, corresponding *p* value < 2.02e-05, Supplementary Table S3); and borderline significant for P (3 CpG sites, FDR = 0.06, corresponding *p* value < 4.30e-07, Supplementary Table S4). Only one CpG was found to show genome-wide significance for menstrual cycle (1 CpG site, cg08916385 on chromosome 4 near gene GNRHR, *p* = 5.09e-10, FDR = 2.47e-04). Manhattan plots for E2, PRL and P are shown in Supplementary Figure S1B, S1C and S1D respectively. In Supplementary Figure S1C, CpGs in the HLA (human leukocyte antigen) region of chromosome 6 are highly associated with PRL in PCOS patients. This is more clearly illustrated by the Manhattan plot for chromosome 6 with the HLA region highlighted with red colour (Figure 2). No genome-wide significant association was found for the other clinical features.

Genes linked to CpGs in Supplementary Tables S2–S4 were submitted to GSEA to look for gene-sets enriched. Significant pathways were found for E2 (2 pathways) and PRL (10 pathways) (Table 3) with no significantly enriched pathways for progesterone. The 2 significant functional pathways for E2 include steroid hormone biosynthesis and metabolism of xenobiotics by cytochrome P450 while the 10 pathways enriched for PRL are dominated by immunity and inflammation processes which largely overlap with the pathways in Table 2.

**Table 3:** The functional pathways enriched (FDR < 0.05) by genes linked to CpG sites in Supplementary Tables S2–S4

Gene Set Name [# Genes (K)]	Description	# Genes in Overlap (k)	*p*-value	FDR (*q*-value)
**Estradiol**				
KEGG:STEROID HORMONE BIOSYNTHESIS [55]	Steroid hormone biosynthesis	3	3.64 e^−5^	4.84 e^−2^
KEGG:METABOLISM OF XENOBIOTICS BY CYTOCHROME P450 [70]	Metabolism of xenobiotics by cytochrome P450	3	7.51 e^−5^	4.99 e^−2^
**Prolactin**				
KEGG: ALLOGRAFT REJECTION [38]	Allograft rejection	3	3.52 e^−5^	2.43 e^−2^
KEGG: GRAFT VERSUS HOST DISEASE [42]	Graft-versus-host disease	3	4.76 e^−5^	2.43 e^−2^
KEGG: TYPE I DIABETES MELLITUS [44]	Type I diabetes mellitus	3	5.48 e^−5^	2.43 e^−2^
KEGG: AUTOIMMUNE THYROID DISEASE [53]	Autoimmune thyroid disease	3	9.59 e^−5^	2.57 e^−2^
REACTOME: ENDOSOMAL VACUOLAR PATHWAY [9]	Genes involved in Endosomal/Vacuolar pathway	2	9.64 e^−5^	2.57 e^−2^
REACTOME: NEF MEDIATED DOWNREGULATION OF MHC CLASS I COMPLEX CELL SURFACE EXPRESSION [10]	Genes involved in Nef mediated downregulation of MHC class I complex cell surface expression	2	1.2 e^−4^	2.67 e^−2^
REACTOME: INTERFERON SIGNALING [159]	Genes involved in Interferon Signaling	4	1.46 e^−4^	2.67 e^−2^
REACTOME: INTERFERON GAMMA SIGNALING [63]	Genes involved in Interferon gamma signaling	3	1.61 e^−4^	2.67 e^−2^
KEGG: VIRAL MYOCARDITIS [73]	Viral myocarditis	3	2.49 e^−4^	3.32 e^−2^
KEGG: ENDOCYTOSIS [183]	Endocytosis	4	2.5 e^−4^	3.32 e^−2^

## DISCUSSION

Although there have been sizable genome-wide studies reporting significant associations between genetic variations and PCOS, [5, 6, 14–17] the regulatory patterns in the molecular pathogenesis of PCOS has been, to date, rarely investigated with only a handful epigenomic studies performed on small sample sizes [10–13]. We have conducted a larger EWAS on DNA methylation in whole blood of 30 PCOS patients and 30 controls. In contrast to previous studies, we engaged a stringent adjustment for multiple testing in our EWAS and were able to identify multiple CpGs reaching genome level significance for their association with PCOS or with clinical features in PCOS patients. Meanwhile, results from our single site analysis also implicate biological pathways that either reconfirming previous studies or representing novel findings.

Although our genome-wide epigenetic profiling was targeted at whole blood DNA, results are however surprisingly highly consistent with a previous study using ovarian tissue [12]. Among the top ten significantly enriched pathways in this study (Table 2), six overlap with the pathways found by Wang et al. [12]. Likewise, in their list of top ten most significant pathways, five can be found in our 22 functional pathways listed in Table 2. It is even more interesting to see that, there are four pathways, i.e. viral myocarditis, allograft rejection, graft versus host disease and type I diabetes mellitus, appearing consistently in the top ten pathways from both studies although each targeting at a very different tissue type (whole blood versus ovarian tissue). While these results are strongly confirmatory and supportive, the high conformity also provides further evidence that the easy-to-access whole blood could serve as a useful surrogate to hard-to-access tissues like ovary to enable non-invasive large scale epigenetic studies on human diseases [18].

Many of the significant pathways in Table 2 are enriched by genes pertaining to immunity (e.g. adaptive immune system, class I MHC mediated antigen processing and presentation), or biological pathways directly related to certain diseases including inflammatory diseases (viral myocarditis, asthma, Leishmania infection), autoimmune diseases (type 1 diabetes mellitus, systemic lupus erythematosus, autoimmune thyroid disease) and immune reaction (allograft rejection, graft versus host disease). The predominant involvement of immunity-related biological pathways emphasizes the crucial role of inflammation and immune reaction in the pathogenesis of PCOS. By comparing the nationwide Danish population of PCOS with a large control group, Glintborg et al. [19] recently reported a significantly increased prevalence for diseases such as diabetes, thyroid disease and asthma. In the literature, high prevalence of autoimmune thyroiditis was also observed in PCOS patients [20–24]. We think that our EWAS results based on DNA methylation profiling provide strong molecular evidence for the epidemiological findings based on clinical data. Overall, both epigenetic and epidemiological data suggest a possible autoimmune basis in the pathogenesis of PCOS.

With interest in the genome-wide distribution of PCOS related CpGs, we calculated the proportion of CpGs in Supplementary Table S1 for their genomic locations (open sea, shelf, shore, island) among hyper- and hypo-methylated CpGs (Supplementary Figures S2A, S2B) and compared them with corresponding proportions in all CpGs on the Illumina 450 K array (Supplementary Figure S2C). Compared with the whole array, the distribution of genomic location for the CpGs in Supplementary Table S1 was significantly different characterized by high proportions of hypermethylated CpGs on both north and south shelves, a low proportion of hypermethylation on the island, and a high proportion of hypomethylated CpGs in the open sea representing isolated CpGs in the genome. The implication of differential genomic distribution of hyper- and hypo-methylated CpGs in the transcriptional regulation of PCOS requires further investigation.

Women with PCOS demonstrate markedly clinical heterogeneity with the commonly associated features neither uniform nor universal [25–26]. Recently, the molecular basis underlying the heterogeneous clinical manifestations of PCOS has been investigated using high-throughput omics approaches and reported molecular biomarkers for metabolic heterogeneity [27]. We point out that their reported findings were based on statistical testing without correction for multiple testing thus missing an important procedure in analysing very high dimensional omics data. Based on genomic DNA methylation profiles measured in our PCOS patients, we were able to conduct association analysis of DNA methylation with multiple clinical features including metabolic parameters and reported significant findings after strict adjustment for multiple testing. Although no genome-wide significant results were found for correlating DNA methylation with any of the metabolic features (BMI, IRI, IRI2, GLU, GLU2, HOMA-IR), highly significant epigenetic associations were observed in our PCOS subjects on multiple reproductive hormones including E2 (Supplementary Table S2), PRL (Supplementary Table S3), and progesterone (Supplementary Table S4). From Table 1, we see that the three hormones have comparable mean levels in PCOS patients and healthy controls. However, for the two hormones with large number of significant CpGs, i.e. E2 (87 CpGs, Supplementary Table S2) and PRL (199 CpGs, Supplementary Table S3), their hormone levels in the blood displayed larger dispersions in PCOS samples as compared with the controls. In Table 3, the methylation regulated pathways for E2 include steroid hormone biosynthesis and metabolism of xenobiotics by cytochrome P450. The two significant biological pathways for E2 reveal, for the first time, the differential regulation in the synthesis of reproductive hormone and in drug metabolism [28] by DNA methylation mechanism in PCOS patients.

Perhaps the most important and novel finding in this study is the genome-wide significant patterns of DNA methylation in association with prolactin level in our PCOS group (Supplementary Table S3). Nearly all of the 10 functional pathways significantly enriched by GSEA (Table 3) are involved in immune function and immune-mediated inflammatory conditions. The strong involvement of immune system in the epigenetic regulation of PRL under PCOS condition is further illustrated by Figure 2 where a remarkably distinct pattern of association is shown in the region of major histocompatibility complex (MHC) on band 6p21.3 of the short arm of chromosome 6, a region harbouring the human leukocyte antigen (HLA) genes (marked in red in Figure 2). Diaz et al. [29] recently summarized the multiple actions of PRL unrelated to reproduction including its role in the immune system in promoting proliferation and in inhibiting apoptosis that could help to maintain the appropriate number of immune cells in physiological conditions and to maintain immune tolerance. Meanwhile, clinical studies failed to associate higher levels of PRL with PCOS [30] and suggested that PCOS and hyperprolactinemia are two distinct entities [31] although low prolactin can be a metabolic risk marker in PCOS patients [32]. Based on these results, one could assume that our observed significant association between DNA methylation and PRL levels could be a phenomenon independent of PCOS and thus should be also observable in non-PCOS subjects. To validate the assumption, we conducted a EWAS on DNA methylation and PRL levels in the 30 control samples of this study. No CpG site was significantly associated with PRL levels in the healthy controls suggesting that the significant association between methylation and PRL is a conditional result only observable in PCOS patients. Although our conclusion requires further validation, it already provides novel suggestive evidence in linking differential DNA methylation and immune responses with PRL regulation in PCOS samples.

It has been suggested that epigenetics may be involved in the regulation of endometrial gene expression during the menstrual cycle in healthy individuals [33]. Our study provides new data on DNA methylation and menstrual cycle in PCOS patients. Although only one CpG (cg08916385) was found, its significance level remained extremely high even at genome level after adjustment for multiple testing. Most importantly, the CpG is located within 1500 bps of the transcription start site (TSS) in the promotor region of gonadotropin-releasing hormone receptor (GNRHR) gene on chromosome 4. This gene encodes the receptor for type 1 gonadotropin-releasing hormone. The gene is expressed on the surface of pituitary gonadotrope cells as well as lymphocytes, breast, ovary, and prostate. After binding of gonadotropin-releasing hormone, the receptor associates with G-proteins that activate a phosphatidylinositol-calcium second messenger system. Activation of the receptor ultimately causes the release of gonadotropic luteinizing hormone (LH) and follicle stimulating hormone (FSH). Our result, for the first time, points to the important role of DNA methylation mediated epigenetic regulation in controlling menstrual cycle in PCOS patients which could impact individualized treatment and management of the disease.

In conclusion, we have identified a substantial number of CpGs differentially methylated in the whole blood samples of PCOS patients and healthy controls, highly consistently replicating biological pathways extensively implicated in immunity and immunity-related inflammatory conditions that were differentially regulated in the DNA methylome of ovarian tissue from PCOS women. Most importantly, our genome-wide DNA methylation profiling focusing on PCOS patients revealed a large number of CpG sites and their enriched functional pathways significantly associated with diverse clinical features (levels of prolactin, estradiol, progesterone and menstrual cycle) that could serve as novel molecular basis of clinical heterogeneity observed in PCOS women.

## MATERIALS AND METHODS

### The study samples

Sample collection was conducted at the Centre of Reproductive Medicine, Linyi People's Hospital, Shandong, China. First, 30 patients aged from 22 to 33 years were recruited from patients diagnosed as PCOS according to the 2003 revised diagnostic criteria of Rotterdam consensus [34]. Based on the age and BMI of the 30 PCOS patients, 30 controls aged from 23 to 32 years were then recruited from healthy females of reproductive age who volunteered to participate. All participants were free from medication and hormone therapy. A written informed consent was obtained from each participant. All experiments were conducted according to the principles of the Declaration of Helsinki. The research was approved by the Reproductive Ethics Committee of Linyi People's Hospital.

### Clinical biochemistry and reproductive hormone

From each participant, blood was taken from antecubital venous for blood biochemical test following routine protocol and meanwhile the blood for DNA methylation analysis was immediately stored under −80°C at the central laboratory of Linyi People's Hospital. Fasting immunoreactive insulin (IRI) and immunoreactive insulin at 2 hours after ingestion of 75 gram dextrose (IRI2) were assayed by radioimmunoassay (Beckman Coulter, Inc., Suzhou, China). Measurements on reproductive hormones i.e. luteinising hormone (LH), follicle stimulating hormone (FSH), estradiol (E2), total testosterone (TST), prolactin, progesterone, and thyroid stimulating hormone (TSH) were determined by direct chemiluminescence immunoassay (Siemens Healthcare Diagnostics Inc; East Walpole, Massachusetts, U.S.A) according to the manufacturer's instructions. Serum fasting blood-glucose (GLU) and GLU at 2 hours after ingestion of 75 gram dextrose (GLU2) were determined by the oxygen electrode method (Beckman Coulter, Inc., Suzhou, China). Homeostatic model assessment IR (HOMA-IR) was calculated by the equation HOMA-IR = GLU*IRI/22.5.

### Genome-wide DNA methylation profiling

Genome-wide DNA methylation level was measured using the Illumina's Infinium HumanMethylation450 Beadchip assay (Illumina, San Diego, CA, USA) at CapitalBio Corporation (http://www.capitalbio.com), a certified Illumina service provider in Beijing, China. The array interrogates over 480,000 CpG sites across and beyond gene and CpG island regions in the human genome. All laboratory work for the assay was performed according to the manufacturer's instructions. Data normalization was done using the free R package *minfi* which employs the quantile normalization [35]. At each CpG site, DNA methylation level was summarized by calculating a methylation “beta” value defined by the Illumina's formula as β = M/(M + U + 100) where M and U are signal intensities measured by the methylated and unmethylated probes at a CpG site. Probe quality was controlled by the detection *p* value calculated using *minfi*. A β value with its assigned detection *p* value > 0.01 was treated as missing. CpG sites with more than 5% missing data across the samples were dropped from the subsequent analysis. Based on the DNA methylation data measured in whole blood of each sample and published cell-type-specific DNA methylation data, the package *minfi* estimated blood cell composition in each individual for 6 blood cell types: CD8T, CD4T, natural killer cell, B cell, monocyte, and granulocyte. The estimated cell type proportion was used by *minfi* to adjust the effects of cell composition on DNA methylation levels in each sample before downstream statistical analysis.

For each individual, DNA methylation levels were measured on a total of 485512 CpG sites across the genome. We first filtered out 728 CpGs with detection *p* value > 0.01 in more than 5% of the overall samples (i.e. 3 samples). Different from other genome-wide analysis that removed all CpGs on sex chromosomes, we only dropped Y-linked CpGs (147 sites) but kept all X-linked CpGs (11229 sites) considering that all our samples are females. A total of 484637 CpG sites were available for subsequent analysis. The raw and normalized DNA methylation data are deposited in the Gene Expression Omnibus database (http://www.ncbi.nlm.nih.gov/geo/; accession no. GSE80468).

### Data analysis

### Clinical data

Comparison of clinical features between PCOS patients and controls was done by the non-parametric Wilcoxon rank sum test (equivalent to the Mann-Whitney test) with consideration that some of the measurements (e.g. menstrual cycle) may not follow the normal distribution. Likewise, dispersion of clinical data was described by calculating the 2.5% and 97.5% quantiles.

### Epigenetic data

For each CpG site, statistical association of DNA methylation with clinical features was tested using the *dmpFinder* function provided by the free R package *minfi* with type of analysis specified as “categorical” for PCOS status (1 for cases and 0 for controls) and as “continuous” for anthropometric and clinical measurements. Continuous measurements were tested with linear regression, while an *F*-test was used for categorical features (here PCOS status, equivalent to a *t*-test), both performed on the logit transformation of the methylation β values. Multiple testing was adjusted by calculating the false discovery rate (FDR) using the Benjamini–Hochberg method [36]. Genome-wide significance in differential methylation was defined as FDR < 0.05.

### Biological pathway analysis

To identify biological pathways differentially regulated by DNA methylation, we used the Gene-Set Enrichment Analysis (GSEA) which is a bioinformatics tool for determining whether an a priori defined set of genes shows statistically significant, concordant differences between two biological states (http://software.broadinstitute.org/gsea/index.jsp) [37]. Based on a collection of the Molecular Signatures Database (MSigDB) of GSEA, we computed overlaps between our genes linked to significant CpGs identified in our analysis and gene sets in MSigDB. Statistical significance of the overlap with each gene set in MSigDB is obtained from the hypergeometric distribution of number of overlapping genes (k), number of genes in the query set (n), total number of genes in a MSigDB set (K) and number of all known genes (N).

$$p\;=\;1\;-\;\sum_{i=0}^{k-1}\left(\begin{array}{c} K\\ i \end{array}\right)\left(\begin{array}{c} N\;-\;K\\ n\;-\;i \end{array}\right)/\left(\begin{array}{c} N\\ n \end{array}\right)$$

Correction for multiple testing was done by calculating the false discovery rate according to Benjamini and Hochberg [36].

### Software packages

All statistical analyses were conducted under the R software environment for statistical computing and graphics (https://www.r-project.org/). DNA methylation data were analysed using the R package *minfi* (http://bioconductor.org/packages/release/bioc/html/minfi.html) [35] for raw data preprocessing (quality control and normalization) and for statistical analysis.

## SUPPLEMENTARY MATERIALS FIGURES AND TABLES


