# Supplementary material for: Differential DNA methylation patterns of polycystic ovarian syndrome in whole blood of Chinese women
Source: Oncotarget. 2016 May 12;8(13):20656–66. doi: 10.18632/oncotarget.9327 (PMC5400534; doi:10.18632/oncotarget.9327)
Supplement: Supplementary file 1 [file oncotarget-08-20656-s001.pdf]

Differential DNA methylation patterns of polycystic ovarian syndrome in whole blood of Chinese women

Supplementary Materials

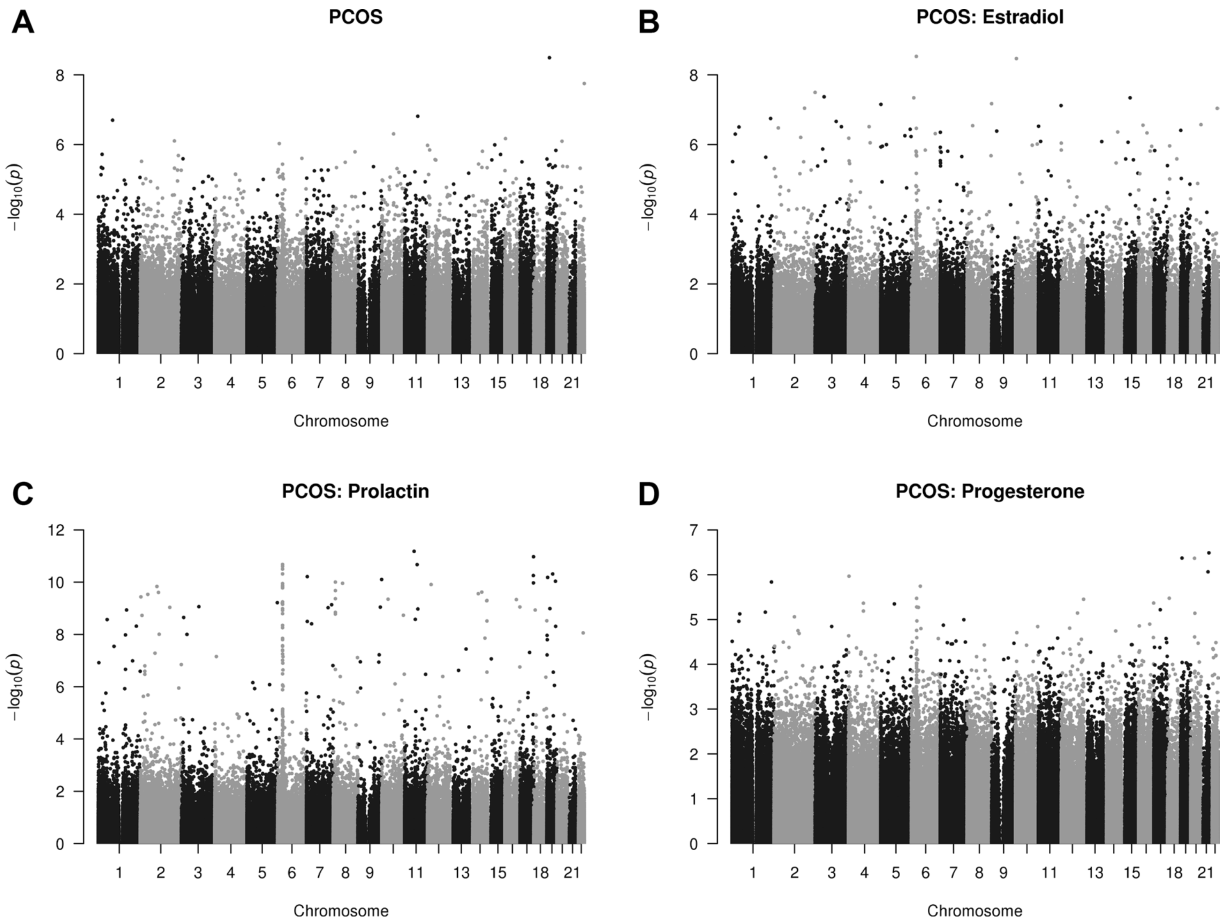

Supplementary Figure S1: Manhattan plots for the negative log10-transformed p values from EWAS on PCOS (A), and EWAS in PCOS patients on E2 (B), prolactin (C) and progesterone (D) plotted against genomic location (Mb) for each CpG. The distinct pattern for prolactin on chromosome 6 is further displayed by a chromosome-wised Manhattan plot in Figure 2.

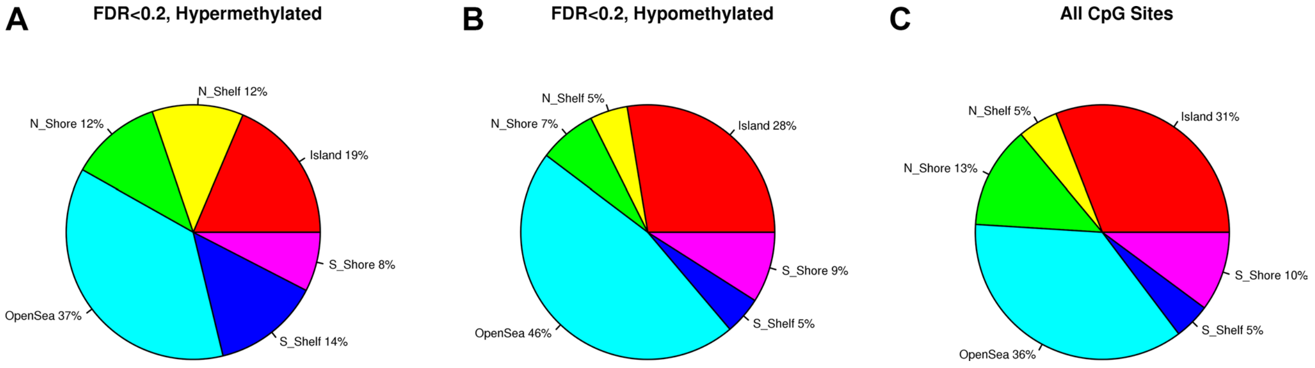

Supplementary Figure S2: Pie plots for the distribution of CpGs associated with PCOS status for hypermethylated (A), hypomethylated (B) CpGs with FDR < 0.2, and for all CpGs analysed on the array (C).

Supplementary Table S1: CpGs associated with PCOS

CPG	meanPcos	meanControl	Diff	pval	qval	chr	pos
cg23647968	0.847061568	0.820176263	0.026885304	3.22E–09	0.001471148	chr19	15051936
cg09456760	0.755388715	0.717660252	0.037728463	1.77E–08	0.004043544	chr22	51206645
cg03197935	0.913973672	0.887855623	0.026118049	1.54E–07	0.022869	chr11	77885410
cg10827434	0.698378549	0.668805774	0.029572774	2.01E–07	0.022869	chr1	84944976
cg07921144	0.777264189	0.799479219	−0.022215029	4.95E–07	0.043882351	chr10	70184359
cg04737885	0.87137745	0.815687391	0.055690059	6.76E–07	0.043882351	chr16	4014095
cg08123608	0.830746535	0.811007865	0.01973867	7.88E–07	0.043882351	chr2	201927099
cg19110428	0.622633757	0.569931651	0.052702105	8.02E–07	0.043882351	chr20	30324912
cg01679528	0.660663744	0.634123913	0.026539831	9.37E–07	0.043882351	chr6	10430966
cg06030190	0.151441802	0.168791335	−0.017349534	1.02E–06	0.043882351	chr15	43029558
cg20454537	0.850726081	0.826026481	0.0246996	1.06E–06	0.043882351	chr12	822286
cg21472448	0.715554946	0.75283076	−0.037275814	1.43E–06	0.046226847	chr12	13156131
cg00703849	0.813809	0.775001563	0.038807437	1.47E–06	0.046226847	chr19	53229766
cg04224092	0.909150343	0.888050429	0.021099914	1.56E–06	0.046226847	chr14	77247777
cg12154749	0.766643613	0.791216125	−0.024572511	1.59E–06	0.046226847	chrX	103176599
cg17820495	0.59077952	0.627429481	−0.036649962	1.62E–06	0.046226847	chr8	128844842
cg00165619	0.623362646	0.655355831	−0.031993185	1.91E–06	0.048820546	chr1	22903087
cg24133724	0.590950244	0.61122771	−0.020277466	1.93E–06	0.048820546	chr15	77271602
cg13131061	0.79096052	0.766329313	0.024631207	2.07E–06	0.049605365	chr2	225268095
cg18368637	0.626520673	0.657858413	−0.031337741	2.49E–06	0.0497538	chr6	144179905
cg04475307	0.817303343	0.777831685	0.039471658	2.56E–06	0.0497538	chr3	9694905
cg18341969	0.138039172	0.1628806	−0.024841428	2.61E–06	0.0497538	chr19	571802
cg25928510	0.852539891	0.840073508	0.012466383	2.70E–06	0.0497538	chr12	45749440
cg07359604	0.74746872	0.762160862	−0.014692142	2.76E–06	0.0497538	chr15	25415763
cg14217534	0.1085838	0.097287356	0.011296444	2.79E–06	0.0497538	chr12	49525474
cg25494151	0.110855693	0.136960392	−0.026104698	3.04E–06	0.0497538	chr2	7006339
cg07940280	0.841908487	0.818550415	0.023358071	3.14E–06	0.0497538	chr17	11054037
cg18227776	0.806633573	0.828776695	−0.022143122	3.23E–06	0.0497538	chr8	71063694
cg05138082	0.878508903	0.861473428	0.017035475	3.63E–06	0.0497538	chr14	54392087
cg22179059	0.868150466	0.841396198	0.026754268	3.66E–06	0.0497538	chr6	29714945
cg02896705	0.609246982	0.588930399	0.020316583	3.76E–06	0.0497538	chr19	18112042
cg25493545	0.102766653	0.119669235	−0.016902582	3.93E–06	0.0497538	chr19	10812419
cg15985418	0.914919536	0.905735814	0.009183722	4.13E–06	0.0497538	chr19	49173732
cg01982835	0.770527939	0.810153243	−0.039625303	4.21E–06	0.0497538	chr20	34995278
cg07645718	0.09100693	0.110580173	−0.019573242	4.30E–06	0.0497538	chr20	61493192
cg23281369	0.102672743	0.114850299	−0.012177556	4.30E–06	0.0497538	chr9	91933285
cg07240000	0.146678451	0.164878525	−0.018200074	4.53E–06	0.0497538	chr1	17878538
cg25966751	0.547743559	0.565402519	−0.01765896	4.54E–06	0.0497538	chr14	74098320
cg14266436	0.883222741	0.876461954	0.006760787	4.57E–06	0.0497538	chr19	34302242
cg08092966	0.871948537	0.888617428	−0.016668891	4.59E–06	0.0497538	chr8	19009591
cg10281977	0.284819895	0.353605549	−0.068785654	4.61E–06	0.0497538	chr14	105070864
cg14376110	0.678155355	0.655080917	0.023074439	4.77E–06	0.0497538	chr2	202354859
cg15788059	0.894690996	0.879478165	0.015212831	4.79E–06	0.0497538	chr22	26961237
cg10176185	0.861428543	0.842802362	0.01862618	4.80E–06	0.0497538	chr1	25894479
cg10715637	0.16788488	0.206197918	−0.038313038	4.91E–06	0.0497538	chr10	90751534

cg14582248	0.743820441	0.778120355	−0.034299913	5.19E–06	0.049815898	chr6	5066984
cg21821755	0.930495223	0.922148033	0.00834719	5.39E–06	0.049815898	chr7	88315898
cg12046414	0.084512498	0.100154771	−0.015642273	5.40E–06	0.049815898	chr2	220379199
cg18356974	0.322261855	0.363458605	−0.04119675	5.40E–06	0.049815898	chr7	128809100
cg19838074	0.894967865	0.88852596	0.006441905	5.46E–06	0.049815898	chr2	242149865
cg09857761	0.84971994	0.862956676	−0.013236736	5.60E–06	0.049846644	chr16	815553
cg03881524	0.777887088	0.79596786	−0.018080772	5.68E–06	0.049846644	chr7	47165287
cg26241240	0.615258685	0.650227232	−0.034968547	6.08E–06	0.051739581	chr11	60893661
cg05175353	0.951897702	0.939760392	0.01213731	6.19E–06	0.051739581	chr6	596127
cg19212550	0.605908766	0.571054158	0.034854608	6.24E–06	0.051739581	chr10	101767908
cg14857063	0.932732102	0.916261482	0.01647062	6.65E–06	0.05416591	chr13	110763402
cg20535253	0.900829218	0.875681243	0.025147975	7.06E–06	0.054866312	chr4	122875768
cg14833935	0.908389292	0.917712972	−0.00932368	7.08E–06	0.054866312	chr6	31927895
cg23843045	0.723134234	0.694576411	0.028557824	7.18E–06	0.054866312	chr4	123297318
cg16883533	0.127965545	0.139096537	−0.011130992	7.23E–06	0.054866312	chr12	7079667
cg00953309	0.889825126	0.876066163	0.013758964	7.34E–06	0.054866312	chr1	16460784
cg24466610	0.931872954	0.92261223	0.009260725	7.98E–06	0.058696288	chr11	433702
cg03413208	0.925994669	0.936343406	−0.010348737	8.20E–06	0.058977974	chr3	160879619
cg06925530	0.839396615	0.85047034	−0.011073725	8.27E–06	0.058977974	chr6	16347397
cg07957471	0.952986407	0.94381576	0.009170647	8.60E–06	0.058989014	chr10	49658574
cg03349251	0.796787769	0.757276657	0.039511112	8.62E–06	0.058989014	chr6	10832472
cg23457357	0.736802767	0.705566711	0.031236056	8.71E–06	0.058989014	chr6	31148748
cg17886715	0.682407193	0.716932708	−0.034525516	8.79E–06	0.058989014	chr1	247269490
cg06096916	0.880015471	0.865146268	0.014869203	9.17E–06	0.059552749	chr7	1989032
cg01381374	0.537326866	0.559360672	−0.022033806	9.46E–06	0.059552749	chr7	93474158
cg00415763	0.690788081	0.662532959	0.028255122	9.57E–06	0.059552749	chr16	66984264
cg24609304	0.846329132	0.862898498	−0.016569365	9.62E–06	0.059552749	chr2	32483287
cg08961047	0.884492596	0.868176909	0.016315687	9.65E–06	0.059552749	chr6	34395138
cg27567135	0.662144319	0.67931311	−0.017168791	9.71E–06	0.059552749	chr17	53095518
cg25867545	0.301429029	0.319451226	−0.018022197	9.79E–06	0.059552749	chr3	182880776
cg18112295	0.1069577	0.120411242	−0.013453541	9.96E–06	0.059810246	chr5	96519566
cg26547526	0.835092431	0.815970167	0.019122264	1.04E–05	0.060853658	chr10	2666743
cg22447396	0.589553583	0.56848849	0.021065094	1.05E–05	0.060853658	chr1	153966266
cg12935979	0.563954749	0.6024864	−0.038531651	1.06E–05	0.060853658	chr4	147559648
cg03889810	0.882511948	0.854603633	0.027908315	1.07E–05	0.060853658	chr7	97884367
cg08676905	0.1113959	0.128854391	−0.017458491	1.09E–05	0.061397533	chr10	6019609
cg25059588	0.859962398	0.875666772	−0.015704373	1.11E–05	0.061864999	chr2	220286185
cg04181696	0.843863113	0.857833881	−0.013970768	1.15E–05	0.062975854	chr2	60981948
cg10582639	0.37162268	0.344186355	0.027436325	1.17E–05	0.063358016	chr11	121526840
cg04216796	0.880067949	0.865073597	0.014994352	1.18E–05	0.063358016	chr3	128770015
cg19471725	0.831534764	0.798268129	0.033266635	1.20E–05	0.063621771	chr3	126113344
cg07809831	0.877661991	0.858000951	0.019661039	1.24E–05	0.063809485	chr16	48428016
cg22375623	0.760500643	0.73957915	0.020921493	1.24E–05	0.063809485	chr15	78444981
cg26756396	0.768880221	0.734714467	0.034165754	1.24E–05	0.063809485	chr19	7459296
cg17445987	0.128754538	0.14673055	−0.017976012	1.27E–05	0.064494536	chr19	10305305
cg24005743	0.480722913	0.49615575	−0.015432837	1.32E–05	0.065592546	chr11	32112810
cg15068487	0.170377729	0.144147787	0.026229942	1.33E–05	0.065592546	chr2	15701235

cg11150068	0.10474812	0.120414803	−0.015666684	1.34E–05	0.065592546	chr10	111683422
cg06820859	0.854018553	0.835942886	0.018075666	1.37E–05	0.065592546	chr13	21564784
cg16275967	0.133182338	0.118376551	0.014805787	1.37E–05	0.065592546	chr1	161172255
cg12582138	0.880035182	0.873080586	0.006954597	1.38E–05	0.065592546	chr17	48183477
cg14151995	0.787593153	0.761727289	0.025865864	1.41E–05	0.066081594	chr11	17793529
cg09675820	0.89926811	0.890168202	0.009099908	1.42E–05	0.066081594	chr17	77997453
cg02070290	0.912873615	0.898606917	0.014266699	1.46E–05	0.067508429	chr17	68071397
cg05844366	0.603847168	0.639590887	−0.03574372	1.49E–05	0.068106268	chr10	91738713
cg10811509	0.654476312	0.674357223	−0.019880911	1.53E–05	0.068840217	chr19	27739313
cg03292388	0.236203977	0.199499189	0.036704788	1.55E–05	0.068840217	chr4	8594514
cg25951580	0.102865129	0.13032228	−0.027457151	1.55E–05	0.068840217	chr19	58962937
cg03983808	0.654200764	0.634636679	0.019564084	1.58E–05	0.069292916	chr10	43133352
cg09362956	0.892909606	0.919567918	−0.026658312	1.64E–05	0.071385779	chr22	32749962
cg04171308	0.099404775	0.109135612	−0.009730837	1.69E–05	0.072698667	chr12	112856542
cg06832605	0.414216957	0.435915753	−0.021698796	1.72E–05	0.073509104	chr20	26319303
cg27105914	0.646209477	0.611742969	0.034466508	1.75E–05	0.073509104	chr17	76445798
cg08862717	0.595382958	0.562576465	0.032806493	1.77E–05	0.073509104	chr2	101763598
cg09767616	0.630652754	0.653749114	−0.023096359	1.77E–05	0.073509104	chr19	58115732
cg05825127	0.831425971	0.846937081	−0.015511109	1.81E–05	0.073687248	chr10	121398040
cg13663416	0.88289499	0.873083114	0.009811875	1.82E–05	0.073687248	chr17	4868062
cg12798564	0.541554718	0.570952523	−0.029397805	1.83E–05	0.073687248	chr3	75263641
cg01403307	0.83509638	0.849446794	−0.014350414	1.86E–05	0.074407588	chr10	134303076
cg18005896	0.824548715	0.842051054	−0.017502339	1.88E–05	0.074407588	chr6	168948135
cg19955849	0.849632484	0.827016688	0.022615796	1.90E–05	0.074713136	chr4	140095286
cg15209934	0.553235794	0.517423528	0.035812267	1.93E–05	0.074974795	chr19	52800385
cg12216772	0.693528313	0.656146717	0.037381596	1.95E–05	0.074974795	chr10	46164062
cg20731100	0.731801833	0.705881319	0.025920514	1.98E–05	0.074974795	chr7	47298162
cg20646500	0.555932874	0.49458779	0.061345084	2.01E–05	0.074974795	chr6	42536105
cg24907970	0.054835867	0.042257018	0.012578848	2.01E–05	0.074974795	chr5	65018155
cg02454501	0.089473122	0.123292242	−0.03381912	2.02E–05	0.074974795	chr11	8285505
cg18249414	0.892823075	0.881788203	0.011034872	2.02E–05	0.074974795	chr15	101626335
cg24688939	0.924756676	0.918207878	0.006548798	2.07E–05	0.076007231	chr21	47545588
cg04252689	0.863876618	0.843456801	0.020419817	2.10E–05	0.076007231	chr19	52210193
cg24148757	0.58260589	0.610485414	−0.027879524	2.10E–05	0.076007231	chr1	112942995
cg05523455	0.163006047	0.176354811	−0.013348763	2.13E–05	0.076678425	chr9	136243219
cg06529894	0.896453319	0.878219494	0.018233826	2.16E–05	0.076915707	chr7	76025038
cg13947513	0.91118286	0.899493635	0.011689226	2.23E–05	0.078693823	chr4	172824551
cg15980914	0.72516401	0.759487223	−0.034323212	2.24E–05	0.078693823	chr19	28297092
cg01441105	0.797149637	0.813971394	−0.016821757	2.34E–05	0.081471851	chr22	38469264
cg10318313	0.466300788	0.485136903	−0.018836115	2.40E–05	0.082294307	chr11	3014937
cg08539210	0.165012897	0.196644912	−0.031632014	2.41E–05	0.082294307	chr16	90038463
cg23924911	0.731528552	0.750997943	−0.019469391	2.42E–05	0.082294307	chr19	43200890
cg20368463	0.95120075	0.960552903	−0.009352153	2.45E–05	0.082315057	chr18	77673604
cg14028967	0.691657647	0.70844449	−0.016786844	2.49E–05	0.082315057	chr9	37419444
cg14339397	0.539873517	0.503753511	0.036120007	2.49E–05	0.082315057	chr13	27338713
cg25652781	0.869169207	0.882797774	−0.013628567	2.49E–05	0.082315057	chr4	153586344
cg15653559	0.869892352	0.853737111	0.016155241	2.56E–05	0.083327483	chr6	31597080

cg01016459	0.096064981	0.10888072	−0.012815739	2.57E–05	0.083327483	chr16	53468960
cg12977937	0.708926893	0.637380818	0.071546075	2.58E–05	0.083327483	chr17	54860136
cg01971789	0.940530954	0.929880006	0.010650948	2.59E–05	0.083327483	chr6	28889545
cg08486065	0.924829953	0.918333693	0.00649626	2.75E–05	0.084059122	chr19	3464875
cg13936208	0.795241673	0.78193364	0.013308033	2.76E–05	0.084059122	chr12	121835047
cg11704463	0.852652929	0.838402338	0.014250591	2.78E–05	0.084059122	chr12	124968399
cg25753720	0.925320767	0.941618636	−0.016297869	2.80E–05	0.084059122	chr2	200904751
cg10081115	0.818626549	0.846775784	−0.028149235	2.84E–05	0.084059122	chr2	190686883
cg21400851	0.608521573	0.631992007	−0.023470434	2.84E–05	0.084059122	chr17	73056681
cg11830508	0.887142343	0.87300111	0.014141233	2.85E–05	0.084059122	chr5	179920202
cg17851126	0.83271607	0.819546153	0.013169917	2.87E–05	0.084059122	chr19	10503723
cg18205465	0.445624101	0.415150582	0.030473518	2.87E–05	0.084059122	chr7	45026543
cg01090686	0.650597144	0.670653742	−0.020056598	2.88E–05	0.084059122	chr12	133000028
cg27362115	0.900680499	0.893724938	0.006955562	2.89E–05	0.084059122	chr11	65172423
cg08867267	0.748353177	0.771453616	−0.023100439	2.90E–05	0.084059122	chrX	130033963
cg08757624	0.783982643	0.737026909	0.046955734	2.91E–05	0.084059122	chr7	132677298
cg23044178	0.912266759	0.930382445	−0.018115686	2.92E–05	0.084059122	chr11	12136405
cg09956747	0.716516285	0.666355358	0.050160927	2.93E–05	0.084059122	chr16	31538556
cg18239858	0.825304109	0.843248467	−0.017944358	2.95E–05	0.084059122	chr7	101712363
cg20944521	0.562304635	0.595401006	−0.033096371	2.96E–05	0.084059122	chr14	22218494
cg13430777	0.821041192	0.839451756	−0.018410564	3.05E–05	0.084059122	chr12	8559701
cg01766505	0.867024996	0.837413574	0.029611422	3.06E–05	0.084059122	chr17	25679717
cg14548038	0.790684192	0.762932443	0.027751749	3.08E–05	0.084059122	chr9	140178418
cg03012289	0.838256653	0.816471941	0.021784712	3.10E–05	0.084059122	chr8	110374374
cg24509398	0.632947386	0.594771596	0.03817579	3.10E–05	0.084059122	chr1	28416532
cg00124836	0.97345123	0.967776092	0.005675137	3.11E–05	0.084059122	chr13	111295144
cg10728179	0.783338096	0.803912657	−0.020574561	3.13E–05	0.084059122	chr3	124798370
cg14334147	0.736571158	0.713784184	0.022786974	3.14E–05	0.084059122	chrX	70291925
cg09463656	0.300051547	0.34296842	−0.042916873	3.15E–05	0.084059122	chr17	13505751
cg10342304	0.758145489	0.740930175	0.017215315	3.15E–05	0.084059122	chr17	730170
cg18050139	0.816330122	0.787891922	0.0284382	3.16E–05	0.084059122	chr12	117099208
cg24882220	0.804133267	0.776958574	0.027174693	3.16E–05	0.084059122	chr8	130945857
cg16657578	0.922691265	0.914217212	0.008474053	3.17E–05	0.084059122	chr1	979589
cg24341554	0.437439141	0.464550539	−0.027111398	3.22E–05	0.084874506	chr19	15591178
cg02193513	0.625304144	0.581132014	0.044172131	3.27E–05	0.084912491	chr4	57395012
cg07990541	0.124024793	0.141774067	−0.017749274	3.33E–05	0.084912491	chr17	72744669
cg15394350	0.060527911	0.070069415	−0.009541504	3.34E–05	0.084912491	chr1	92950257
cg08411094	0.850284625	0.82422636	0.026058265	3.39E–05	0.084912491	chr12	12916279
cg16004439	0.857605816	0.837042149	0.020563667	3.40E–05	0.084912491	chr4	165851186
cg24027342	0.771403227	0.756671344	0.014731883	3.40E–05	0.084912491	chr11	108131609
cg00299070	0.807814929	0.769261442	0.038553487	3.41E–05	0.084912491	chr1	237946534
cg00086710	0.275087771	0.233517269	0.041570502	3.42E–05	0.084912491	chr4	89204074
cg03342862	0.79780593	0.811321767	−0.013515837	3.42E–05	0.084912491	chr1	163725217
cg10256304	0.745993182	0.764679371	−0.018686189	3.42E–05	0.084912491	chr11	3497985
cg08199235	0.74834047	0.780343825	−0.032003354	3.44E–05	0.084912491	chr2	131085606
cg18013249	0.807940313	0.821074851	−0.013134538	3.44E–05	0.084912491	chr7	65961473
cg10474284	0.789821018	0.771699153	0.018121865	3.49E–05	0.085319948	chr13	95258374

cg18077418	0.57909044	0.603883011	−0.024792571	3.52E–05	0.085319948	chr4	6921774
cg26668872	0.843506341	0.861000306	−0.017493965	3.52E–05	0.085319948	chr16	3546216
cg13453288	0.730694016	0.707483047	0.023210969	3.55E–05	0.085319948	chr1	53166996
cg19145584	0.693181942	0.712141084	−0.018959142	3.58E–05	0.085319948	chr7	65865556
cg03728457	0.899706482	0.88897664	0.010729841	3.59E–05	0.085319948	chr15	74619595
cg10312190	0.82599479	0.840243318	−0.014248529	3.59E–05	0.085319948	chr11	67749846
cg04791456	0.829337125	0.845947706	−0.016610581	3.63E–05	0.085319948	chr3	57505743
cg16705229	0.860068663	0.832075913	0.02799275	3.63E–05	0.085319948	chr6	30019131
cg17916447	0.847425074	0.864983334	−0.01755826	3.70E–05	0.086402161	chr3	197356641
cg12478793	0.782977954	0.761673813	0.021304141	3.75E–05	0.086402161	chr19	37261338
cg00333020	0.888956661	0.883114863	0.005841798	3.77E–05	0.086402161	chr10	2932299
cg14535019	0.700980511	0.719786788	−0.018806276	3.79E–05	0.086402161	chr4	129433853
cg00395990	0.726873809	0.687104359	0.03976945	3.82E–05	0.086402161	chr11	119054921
cg23237765	0.66829615	0.593000547	0.075295603	3.82E–05	0.086402161	chr7	921845
cg11087438	0.796380638	0.814113439	−0.017732802	3.84E–05	0.086402161	chr17	18302510
cg22118112	0.943997703	0.935691408	0.008306295	3.84E–05	0.086402161	chr20	62657367
cg15108752	0.908500864	0.91898491	−0.010484046	3.86E–05	0.086402161	chr10	12874253
cg08523325	0.795695209	0.80682652	−0.01113131	3.88E–05	0.086402161	chr11	89901450
cg08651894	0.793185172	0.806540627	−0.013355455	3.88E–05	0.086402161	chr1	154832235
cg16987567	0.802796653	0.778753239	0.024043414	3.94E–05	0.087159598	chr1	33790374
cg08219459	0.834161492	0.80823318	0.025928311	3.97E–05	0.08754075	chr16	68734735
cg19831386	0.848348129	0.832409786	0.015938343	4.04E–05	0.088607006	chr14	105235891
cg03047400	0.705855971	0.678258132	0.027597839	4.14E–05	0.090418163	chr14	73496407
cg16976547	0.864363082	0.847459908	0.016903174	4.17E–05	0.090645626	chr15	91433530
cg05014842	0.887675012	0.872895781	0.014779232	4.27E–05	0.092231676	chr2	239225457
cg14028272	0.834228926	0.812118244	0.022110683	4.33E–05	0.093285351	chr1	229533222
cg00808730	0.170552541	0.194600382	−0.024047841	4.37E–05	0.093509213	chr14	93215038
cg09561417	0.547098511	0.569709343	−0.022610832	4.61E–05	0.097323425	chr17	18223010
cg17794261	0.114983969	0.125156923	−0.010172954	4.62E–05	0.097323425	chr16	30583759
cg07728084	0.757667311	0.778301505	−0.020634194	4.64E–05	0.097323425	chr10	21683123
cg10351617	0.868537265	0.861023173	0.007514092	4.68E–05	0.097323425	chr21	47580738
cg27190839	0.713263736	0.683598979	0.029664757	4.69E–05	0.097323425	chr11	33097035
cg01379207	0.765283043	0.781987954	−0.016704912	4.71E–05	0.097323425	chr11	127811604
cg12886731	0.643299559	0.602468219	0.04083134	4.71E–05	0.097323425	chr10	71074462
cg21740507	0.632398615	0.600617574	0.031781041	4.71E–05	0.097323425	chr2	237412985
cg26018312	0.862315911	0.877679354	−0.015363444	4.75E–05	0.097546865	chrX	138529149
cg14999726	0.851061895	0.866081587	−0.015019692	4.78E–05	0.097892863	chr17	31251090
cg14152854	0.873356941	0.859377316	0.013979626	4.81E–05	0.09790196	chr9	138983759
cg26029736	0.264352965	0.317835861	−0.053482897	4.85E–05	0.098043422	chr2	105760375
cg15365536	0.700912855	0.719575082	−0.018662227	4.86E–05	0.098043422	chr6	16266181
cg21678396	0.938105995	0.945385953	−0.007279958	4.88E–05	0.098043422	chr12	133145052
cg18633587	0.928934911	0.913213917	0.015720994	4.91E–05	0.09825211	chr3	48016946
cg07971743	0.665408143	0.683315102	−0.01790696	4.95E–05	0.09825211	chr6	169235842
cg20738202	0.070588115	0.092772392	−0.022184277	4.95E–05	0.09825211	chr11	16759965
cg17972930	0.827098831	0.86843777	−0.04133894	4.99E–05	0.098589552	chr15	50797795
cg12798675	0.539475703	0.509273143	0.03020256	5.07E–05	0.099277506	chr14	102171582
cg13559773	0.803791447	0.786202258	0.017589189	5.09E–05	0.099277506	chr19	48562267

cg08862181	0.715822068	0.69890355	0.016918518	5.13E–05	0.099277506	chr17	10277045
cg24924210	0.148856544	0.167689211	−0.018832666	5.13E–05	0.099277506	chr19	16683266
cg03536022	0.815689344	0.769783416	0.045905928	5.17E–05	0.099277506	chr6	29706879
cg04400496	0.675192577	0.701479575	−0.026286999	5.17E–05	0.099277506	chr12	75057913
cg18185028	0.727060694	0.704087809	0.022972884	5.18E–05	0.099277506	chr3	154042079
cg23906204	0.854982803	0.873503556	−0.018520753	5.22E–05	0.099335577	chr1	161110860
cg21540970	0.333404245	0.366188911	−0.032784666	5.23E–05	0.099335577	chr2	20075083
cg16815991	0.208191394	0.236673622	−0.028482229	5.34E–05	0.100237176	chr12	14133129
cg01730032	0.777333885	0.757279348	0.020054537	5.35E–05	0.100237176	chr4	135248255
cg04630873	0.894049744	0.877555825	0.016493919	5.37E–05	0.100237176	chr10	33171196
cg02234734	0.88847767	0.877079004	0.011398666	5.38E–05	0.100237176	chr1	44882329
cg22313497	0.122395423	0.146144659	−0.023749236	5.38E–05	0.100237176	chr16	730927
cg03251249	0.088632063	0.099124751	−0.010492688	5.45E–05	0.100637777	chr10	69645012
cg01924711	0.929613211	0.920595461	0.00901775	5.46E–05	0.100637777	chr7	86829809
cg01409552	0.578516307	0.69965287	−0.121136563	5.47E–05	0.100637777	chr16	28830997
cg12875892	0.126626335	0.142823235	−0.0161969	5.49E–05	0.100637777	chr1	236030587
cg07217075	0.069075139	0.056349897	0.012725242	5.57E–05	0.101382394	chr20	17511826
cg20385970	0.575650483	0.605720771	−0.030070288	5.58E–05	0.101382394	chr15	25474668
cg08460629	0.871243523	0.888489232	−0.017245709	5.61E–05	0.101382394	chr13	64315383
cg15911493	0.477168159	0.501941381	−0.024773222	5.62E–05	0.101382394	chr14	31529383
cg14941172	0.519590328	0.497168606	0.022421722	5.68E–05	0.102041824	chr19	56733045
cg07790826	0.266803438	0.229403672	0.037399766	5.76E–05	0.102964729	chr11	70049435
cg15422439	0.720777904	0.736304077	−0.015526173	5.79E–05	0.10306306	chr8	70549038
cg13852536	0.76859097	0.779317018	−0.010726048	5.84E–05	0.10306306	chr9	32567045
cg18045461	0.536432694	0.515585458	0.020847236	5.88E–05	0.10306306	chr22	29601862
cg10262775	0.896433491	0.884818487	0.011615004	5.94E–05	0.10306306	chr17	293318
cg04787785	0.150006109	0.134096594	0.015909515	5.95E–05	0.10306306	chr16	19533038
cg18155853	0.952494505	0.943841787	0.008652718	5.96E–05	0.10306306	chr3	169540504
cg27351837	0.181728154	0.200277414	−0.01854926	5.96E–05	0.10306306	chr15	92396247
cg27047750	0.164877306	0.190692567	−0.025815261	5.97E–05	0.10306306	chr19	42365024
cg05516986	0.098426404	0.107185967	−0.008759563	5.98E–05	0.10306306	chr7	158622474
cg20187309	0.795701552	0.760116667	0.035584884	5.99E–05	0.10306306	chr6	26535971
cg12188830	0.076229624	0.063506107	0.012723517	6.05E–05	0.103199491	chr3	57741926
cg11123583	0.903413036	0.925691277	−0.022278241	6.06E–05	0.103199491	chr17	744766
cg15831820	0.572071779	0.541421286	0.030650494	6.06E–05	0.103199491	chr10	88214335
cg09074938	0.910581812	0.897481943	0.013099869	6.08E–05	0.103199491	chr8	144757347
cg02978168	0.371236538	0.432609354	−0.061372817	6.48E–05	0.109237214	chr18	76695123
cg17908719	0.373548024	0.394057166	−0.020509142	6.49E–05	0.109237214	chr3	170155556
cg08441269	0.790749903	0.810827168	−0.020077265	6.56E–05	0.109719883	chr4	53815811
cg13823366	0.800711872	0.820732298	−0.020020426	6.57E–05	0.109719883	chr17	6678929
cg09822423	0.897153445	0.9098573	−0.012703855	6.60E–05	0.109863328	chr1	192509700
cg27016609	0.51860203	0.541574985	−0.022972955	6.73E–05	0.111431704	chr2	48806774
cg21218627	0.800116121	0.784313252	0.015802869	6.80E–05	0.111431704	chr9	132999496
cg12423473	0.965070433	0.957660641	0.007409792	6.82E–05	0.111431704	chr15	45962135
cg26614346	0.600612484	0.572494756	0.028117728	6.84E–05	0.111431704	chr2	231085735
cg09050761	0.764781239	0.720673652	0.044107586	6.85E–05	0.111431704	chr8	17272669
cg14275626	0.865979929	0.828321362	0.037658567	6.87E–05	0.111431704	chr9	135549588

cg22624255	0.151401705	0.181236881	−0.029835176	6.87E–05	0.111431704	chr19	19779476
cg10233416	0.925096194	0.915175692	0.009920502	6.89E–05	0.111431704	chr6	36414541
ch.10.2810236F	0.086571422	0.098740079	−0.012168657	6.96E–05	0.111885981	chr10	130385987
cg02842869	0.766190651	0.785302396	−0.019111745	6.98E–05	0.111885981	chr17	79921131
cg25100475	0.827104827	0.846560076	−0.019455249	6.99E–05	0.111885981	chr1	227750970
cg24163448	0.671811262	0.622587124	0.049224138	7.04E–05	0.112278447	chr9	79013444
cg08119506	0.777809768	0.790953181	−0.013143413	7.09E–05	0.112291486	chr8	117309811
cg26887757	0.857030235	0.840077233	0.016953002	7.09E–05	0.112291486	chr16	70331777
cg12682872	0.890731453	0.904272209	−0.013540756	7.13E–05	0.112291486	chr6	32919427
cg21276022	0.718628088	0.688328845	0.030299243	7.14E–05	0.112291486	chr9	136390236
cg22232695	0.772913078	0.742240786	0.030672292	7.18E–05	0.112412968	chr19	36156356
cg11662609	0.869479457	0.856380687	0.01309877	7.19E–05	0.112412968	chr10	5935006
cg11532725	0.818237382	0.791738472	0.02649891	7.26E–05	0.112748195	chr2	56407143
cg00711000	0.113032405	0.126002813	−0.012970408	7.28E–05	0.112748195	chr2	36824663
cg22699314	0.680829394	0.713626846	−0.032797452	7.29E–05	0.112748195	chr2	61847725
cg08407553	0.775977534	0.75524435	0.020733184	7.40E–05	0.113837378	chrX	70587800
cg00171942	0.77500991	0.740914358	0.034095551	7.44E–05	0.113837378	chr1	34088611
cg09447450	0.810683801	0.824722875	−0.014039073	7.45E–05	0.113837378	chr6	40563644
cg05413325	0.876038742	0.870034873	0.006003869	7.46E–05	0.113837378	chr8	1397936
cg09441501	0.06167644	0.080520804	−0.018844364	7.55E–05	0.114581258	chr11	798350
cg06924976	0.142661736	0.15538222	−0.012720483	7.56E–05	0.114581258	chr4	41753749
cg02793656	0.919143816	0.909575385	0.009568431	7.61E–05	0.114581258	chr15	26869667
cg08047457	0.079202261	0.086825741	−0.00762348	7.64E–05	0.114581258	chr3	50378413
cg21769114	0.076409562	0.085311985	−0.008902423	7.64E–05	0.114581258	chr3	32726526
cg06517813	0.641070603	0.688647356	−0.047576753	7.66E–05	0.114581258	chr1	198553499
cg24037243	0.236332732	0.269391738	−0.033059007	7.69E–05	0.114581258	chr17	8534057
cg20568408	0.601175688	0.617373833	−0.016198145	7.83E–05	0.115744307	chr14	90425050
cg05055833	0.796595094	0.830636407	−0.034041312	7.84E–05	0.115744307	chr15	25459305
cg10464462	0.06141317	0.081253854	−0.019840684	7.84E–05	0.115744307	chr17	33417059
cg26529556	0.143626085	0.158114559	−0.014488474	7.86E–05	0.115744307	chr17	62223225
cg15028479	0.748919269	0.768558512	−0.019639244	7.91E–05	0.115834639	chr1	238081782
cg10151248	0.868342878	0.855010644	0.013332234	7.92E–05	0.115834639	chr11	66639567
cg24850331	0.059936553	0.048043456	0.011893096	7.96E–05	0.115891735	chr12	70132987
cg12315011	0.564403847	0.589589613	−0.025185766	7.98E–05	0.115891735	chr4	161047106
cg17696406	0.802748143	0.816605913	−0.01385777	8.01E–05	0.116053174	chr3	61421175
cg03325407	0.717874697	0.692607567	0.02526713	8.08E–05	0.116513683	chr1	114423726
cg17768026	0.823260587	0.800390228	0.022870359	8.23E–05	0.116513683	chr6	137140006
cg11918172	0.681448091	0.702107408	−0.020659317	8.24E–05	0.116513683	chr2	104914351
cg19282068	0.744951226	0.763311806	−0.01836058	8.24E–05	0.116513683	chr4	3899446
cg15990211	0.084574502	0.095523564	−0.010949063	8.25E–05	0.116513683	chr14	65290093
cg18499294	0.798822721	0.777403351	0.02141937	8.26E–05	0.116513683	chr14	55072950
cg09261992	0.801842012	0.775209271	0.026632741	8.27E–05	0.116513683	chr1	31510528
cg24431909	0.077276749	0.089591629	−0.012314881	8.27E–05	0.116513683	chr19	13030239
cg06703635	0.756449223	0.782676674	−0.026227451	8.28E–05	0.116513683	chr1	201488410
cg07085310	0.840353074	0.858732632	−0.018379558	8.30E–05	0.116513683	chr3	44752404
cg11393407	0.901359806	0.889967646	0.011392161	8.38E–05	0.11724415	chr17	17717239
cg02780400	0.754443422	0.725764543	0.028678879	8.60E–05	0.119939573	chr10	43187469

cg27070729	0.892732458	0.874352017	0.018380441	8.63E–05	0.119939573	chr16	28964954
cg11722990	0.832493352	0.810137873	0.022355479	8.66E–05	0.119939573	chr13	96984206
cg19462712	0.809170321	0.82042916	−0.011258839	8.68E–05	0.119939573	chr19	34744703
cg23397147	0.67223493	0.628868269	0.043366662	8.70E–05	0.119939573	chr17	4063598
cg23088403	0.846300464	0.834523912	0.011776552	8.78E–05	0.120613244	chr7	56434239
cg10550471	0.714036243	0.68483885	0.029197393	8.82E–05	0.120810942	chr7	156239550
cg08983966	0.942715576	0.934048275	0.008667301	8.88E–05	0.121295777	chr17	76798273
cg25922355	0.661013909	0.645082097	0.015931812	8.94E–05	0.121392452	chr7	75830799
cg06919490	0.122102365	0.139903008	−0.017800642	8.95E–05	0.121392452	chr4	166034313
cg03369671	0.108919439	0.12435461	−0.015435171	8.99E–05	0.121392452	chr2	232063176
cg21827031	0.234856198	0.262827636	−0.027971438	9.03E–05	0.121392452	chr11	46958255
cg18612204	0.191112101	0.220258294	−0.029146193	9.04E–05	0.121392452	chr15	65204146
cg24400553	0.792796419	0.7782818	0.014514619	9.05E–05	0.121392452	chr19	1886629
cg01372694	0.585084406	0.601981449	−0.016897043	9.07E–05	0.12140893	chr7	65878352
cg05233128	0.932969933	0.928855312	0.004114621	9.28E–05	0.12357485	chr19	3659669
cg08351269	0.819401254	0.802177059	0.017224195	9.29E–05	0.12357485	chr5	108674169
cg20183778	0.044424028	0.035409146	0.009014882	9.34E–05	0.123605033	chr7	102105344
cg23841538	0.685059982	0.703126857	−0.018066875	9.35E–05	0.123605033	chr2	206804487
cg21787873	0.648987437	0.673313298	−0.024325861	9.43E–05	0.124312224	chr1	23276711
cg01870247	0.84351366	0.816736913	0.026776747	9.49E–05	0.124729121	chr20	31354127
cg13525458	0.15277479	0.169482191	−0.016707401	9.62E–05	0.126006714	chr20	44486289
cg19727641	0.159647817	0.132406515	0.027241302	9.66E–05	0.126006714	chr3	138067606
cg09541468	0.081697249	0.093975914	−0.012278665	9.67E–05	0.126006714	chr11	67211245
cg05305025	0.096664576	0.111789623	−0.015125047	9.78E–05	0.126177178	chr18	20513713
cg18611122	0.238062088	0.269587911	−0.031525823	9.79E–05	0.126177178	chr1	150947896
cg24933247	0.067423658	0.088235361	−0.020811703	9.82E–05	0.126177178	chr19	19729558
cg19631346	0.822769469	0.838855141	−0.016085672	9.85E–05	0.126177178	chr19	22699252
cg23627475	0.580030738	0.610229102	−0.030198364	9.85E–05	0.126177178	chr2	216180626
cg27089675	0.364287029	0.40298956	−0.038702531	9.86E–05	0.126177178	chr10	123838499
cg13976866	0.571105623	0.582139761	−0.011034138	9.90E–05	0.126177178	chr7	128696069
cg09439010	0.555537843	0.579641896	−0.024104053	9.91E–05	0.126177178	chr4	55852467
cg10907356	0.683194137	0.705445627	−0.02225149	9.93E–05	0.126177178	chr2	17830694
cg22866835	0.116068155	0.097137872	0.018930283	0.000100833	0.127467674	chr10	35931235
cg26336773	0.831994323	0.84476837	−0.012774047	0.000100861	0.127467674	chr1	175014805
cg11019069	0.822228465	0.806264006	0.015964459	0.00010202	0.12857553	chr6	28551028
cg24469803	0.10364012	0.121052831	−0.017412712	0.000103298	0.128699513	chr10	79789416
cg25320665	0.862247403	0.875981162	−0.013733759	0.000104023	0.128699513	chr11	102668176
cg11867686	0.276449478	0.240845332	0.035604147	0.000104264	0.128699513	chr11	67169368
cg24029994	0.900955886	0.894616139	0.006339747	0.000104612	0.128699513	chr11	1471410
cg25597531	0.955528639	0.963880064	−0.008351425	0.000104664	0.128699513	chr8	54507339
cg08699341	0.903631067	0.890109187	0.01352188	0.000105176	0.128699513	chr13	114193224
cg27166452	0.875761427	0.859530598	0.016230829	0.000105263	0.128699513	chr6	116592422
cg10504606	0.551496943	0.583711438	−0.032214494	0.000105427	0.128699513	chr17	8464766
cg01044580	0.917680289	0.905649643	0.012030646	0.000105832	0.128699513	chr14	21900820
cg06708683	0.753170924	0.714550443	0.038620481	0.000105925	0.128699513	chr17	42782472
cg16466278	0.678923147	0.704084328	−0.025161181	0.000106146	0.128699513	chr15	43429893
cg07441122	0.733156566	0.692278873	0.040877693	0.000106236	0.128699513	chr16	46824519

cg26737330	0.876772818	0.898818371	−0.022045552	0.000107283	0.128699513	chr4	71200323
cg12518259	0.741883194	0.767687611	−0.025804417	0.000107413	0.128699513	chrX	100668953
cg18437365	0.450230945	0.42082233	0.029408615	0.000107425	0.128699513	chr13	112717484
cg09791009	0.568420683	0.608488405	−0.040067722	0.000107523	0.128699513	chr12	73034008
cg04514834	0.783260648	0.803181656	−0.019921008	0.000107766	0.128699513	chr5	117084231
cg15477139	0.509726998	0.533730423	−0.024003425	0.000107996	0.128699513	chr15	25223633
cg11545863	0.79626493	0.777873188	0.018391742	0.000108107	0.128699513	chr10	102487168
cg11126351	0.892654672	0.880726399	0.011928273	0.000108153	0.128699513	chr14	35594356
cg24985372	0.553590377	0.593840524	−0.040250147	0.000108286	0.128699513	chr2	130741520
cg11415852	0.831114084	0.812166549	0.018947535	0.000108731	0.128699513	chr10	97201765
cg13883186	0.907982637	0.920032249	−0.012049612	0.000108774	0.128699513	chr7	142333186
cg01186613	0.912326746	0.898323971	0.014002776	0.000109012	0.128699513	chr2	44065003
cg02391555	0.795502492	0.776999988	0.018502504	0.000109171	0.128699513	chr16	22016152
cg14847236	0.941374545	0.946264375	−0.004889831	0.000110503	0.12993494	chr4	1656956
cg16736181	0.596615876	0.615899824	−0.019283948	0.000111102	0.130303374	chr10	2249818
cg01795660	0.746307621	0.73074779	0.015559831	0.000112387	0.131309559	chr16	30024074
cg18720905	0.080517206	0.087728414	−0.007211208	0.000113169	0.131309559	chr2	96990858
cg24102266	0.104938535	0.118628902	−0.013690367	0.000113642	0.131309559	chr1	33219584
cg15703426	0.823143874	0.800941579	0.022202295	0.000113857	0.131309559	chr6	14409427
cg26505691	0.041717454	0.031357523	0.010359931	0.000113925	0.131309559	chr16	27461333
cg24517611	0.751476069	0.731739377	0.019736692	0.000114083	0.131309559	chr17	73203081
cg18114913	0.726488873	0.746578929	−0.020090056	0.000114138	0.131309559	chr6	167738342
cg16508522	0.784277349	0.805463565	−0.021186216	0.000114263	0.131309559	chr3	141319423
cg00597801	0.089675813	0.102440205	−0.012764392	0.000115022	0.131690453	chr4	109092918
cg05965444	0.607021453	0.588445083	0.01857637	0.000115171	0.131690453	chrX	134049328
cg24345747	0.610903808	0.643236072	−0.032332265	0.000116449	0.132584494	chr2	87015813
cg02238950	0.610504303	0.625991187	−0.015486883	0.000116535	0.132584494	chr17	1505303
cg16006841	0.694710434	0.502286769	0.192423665	0.000118073	0.134000963	chr5	176797999
cg11625382	0.881817397	0.86112666	0.020690737	0.00011853	0.134053681	chr10	3098271
cg27298394	0.871801047	0.885881242	−0.014080195	0.000118725	0.134053681	chr6	23854222
cg03338185	0.67677363	0.632841965	0.043931665	0.000119023	0.134053681	chr1	16304821
cg20058389	0.858224684	0.865683866	−0.007459182	0.000119295	0.134053681	chr2	196412552
cg14331148	0.151109309	0.175595578	−0.024486269	0.000120163	0.13451832	chr19	47551893
cg11120049	0.742880932	0.708241466	0.034639466	0.000120438	0.13451832	chr14	78104508
cg08938155	0.719084977	0.66165002	0.057434957	0.000120593	0.13451832	chr5	77043612
cg17002428	0.690481683	0.659765813	0.03071587	0.00012238	0.136178225	chr1	3778271
cg18075930	0.723454725	0.742153235	−0.01869851	0.000123149	0.136700634	chr7	30615805
cg14502625	0.277386247	0.306820951	−0.029434704	0.000123569	0.136834533	chr5	33162283
cg07990395	0.689674685	0.708622708	−0.018948022	0.000124244	0.137247967	chrX	138287169
cg03847896	0.616663461	0.567293149	0.049370312	0.000127	0.139657591	chr1	112154295
cg16709512	0.862247887	0.871529312	−0.009281425	0.000127349	0.139657591	chr5	101297174
cg06223797	0.852872314	0.83772026	0.015152055	0.000128087	0.139657591	chr12	95644358
cg24539380	0.845702148	0.831417999	0.014284149	0.000128382	0.139657591	chr17	36896750
cg21651356	0.732738326	0.717552742	0.015185584	0.000128602	0.139657591	chr12	104685539
cg14530856	0.894022296	0.878665453	0.015356843	0.000128879	0.139657591	chr10	778073
cg10162673	0.069091466	0.07787746	−0.008785994	0.000129029	0.139657591	chr3	128598689
cg17610333	0.169580982	0.254126678	−0.084545695	0.000129385	0.139657591	chr6	168107313

cg20844692	0.216780498	0.253280059	−0.036499561	0.000129469	0.139657591	chr3	196230657
cg06819813	0.52928037	0.557530524	−0.028250154	0.000129486	0.139657591	chr15	25471851
cg08962798	0.508121978	0.529435872	−0.021313894	0.00012994	0.139816242	chr5	179106333
cg08047886	0.157354922	0.183794711	−0.026439789	0.000131238	0.140811189	chr11	57425128
cg11964474	0.769144961	0.756355764	0.012789197	0.000131853	0.140811189	chr17	41278655
cg08856378	0.748675917	0.767389851	−0.018713934	0.000132	0.140811189	chr17	560590
cg01084918	0.524504766	0.545080816	−0.02057605	0.000132099	0.140811189	chr1	110576366
cg02150077	0.611809663	0.573189802	0.038619861	0.000132536	0.140947978	chr16	87098542
cg07917842	0.5242867	0.536984348	−0.012697648	0.000133135	0.141256087	chr17	29672644
cg19890858	0.146138331	0.171287258	−0.025148926	0.000134216	0.141478559	chr22	50624742
cg02545885	0.867459065	0.850378947	0.017080118	0.000134261	0.141478559	chr20	16707078
cg04730276	0.872436208	0.848349126	0.024087083	0.000134275	0.141478559	chr2	228586426
cg13118072	0.186077959	0.158350967	0.027726992	0.000134676	0.141573984	chr6	30181194
cg17686236	0.788557574	0.750290488	0.038267086	0.000135893	0.142524501	chr7	6444640
cg09102864	0.12339755	0.136745471	−0.01334792	0.000136417	0.142746129	chr11	68780892
cg02483910	0.865396521	0.851710252	0.013686268	0.000137789	0.143851875	chr7	66055597
cg25228995	0.895665545	0.885349918	0.010315627	0.000138818	0.144595356	chr5	139260468
cg09796583	0.741315641	0.715258958	0.026056683	0.000139185	0.144647348	chr10	73156647
cg18042586	0.607949601	0.58949862	0.018450981	0.000139899	0.144833229	chr8	22288224
cg10124011	0.845958163	0.830640092	0.015318071	0.000140095	0.144833229	chr1	1574521
cg12295617	0.10932344	0.119619923	−0.010296483	0.000140502	0.144833229	chr8	22526663
cg26891211	0.832914451	0.774483673	0.058430778	0.000140634	0.144833229	chr16	17931006
cg20827193	0.085598379	0.09499873	−0.009400352	0.000141249	0.145139318	chr1	109969084
cg12610079	0.598825508	0.622017193	−0.023191684	0.000142567	0.145857777	chr1	162838809
ch.12.2583928F	0.202137293	0.214616233	−0.01247894	0.000142588	0.145857777	chr12	123160012
cg00766964	0.820865998	0.799697434	0.021168564	0.000143551	0.146432468	chr13	24693844
cg07403638	0.168315978	0.14710605	0.021209928	0.000143965	0.146432468	chr18	32870369
cg02023042	0.55577807	0.538433215	0.017344855	0.000144112	0.146432468	chr7	92220071
cg06311795	0.675203836	0.699071213	−0.023867376	0.000144846	0.146770605	chr12	130818957
cg17303578	0.769558609	0.739459911	0.030098699	0.000145089	0.146770605	chr5	128303903
cg08877035	0.118460829	0.132866369	−0.01440554	0.000145826	0.147190605	chr12	62829123
cg02137691	0.786803165	0.769047429	0.017755736	0.000146596	0.147640778	chr4	1805671
cg08740309	0.807201403	0.793148042	0.014053361	0.000148372	0.14770583	chr11	8891157
cg02980650	0.781831378	0.807364633	−0.025533255	0.000148412	0.14770583	chr10	7446012
cg27234786	0.363513656	0.398382883	−0.034869226	0.000148528	0.14770583	chr1	204381404
cg14560311	0.082517869	0.061330632	0.021187237	0.000148678	0.14770583	chr15	34517239
cg25215117	0.889788482	0.880312627	0.009475855	0.000148704	0.14770583	chr17	11461665
cg21613620	0.904841096	0.92293266	−0.018091565	0.000148775	0.14770583	chr13	97927242
cg17384105	0.82733937	0.794912807	0.032426563	0.000148927	0.14770583	chr6	32126949
cg04471375	0.663290583	0.696466498	−0.033175915	0.000149966	0.147996682	chr16	58752165
cg24688909	0.879495475	0.884911809	−0.005416334	0.000150017	0.147996682	chr22	47059079
cg02567751	0.841618302	0.825403011	0.016215292	0.000150193	0.147996682	chr6	39521911
cg18193219	0.121534965	0.137751682	−0.016216717	0.000151692	0.148665941	chr3	53164697
cg26371226	0.883921562	0.869560064	0.014361498	0.000151774	0.148665941	chr6	4259185
ch.10.645342R	0.17644751	0.189977897	−0.013530387	0.00015185	0.148665941	chr10	24771691
cg12819931	0.710822312	0.737801063	−0.026978751	0.000152718	0.149195647	chr10	30663455
cg03392960	0.602166487	0.620989139	−0.018822652	0.000153288	0.149432641	chr8	49823140

cg02058552	0.092269622	0.109420374	−0.017150752	0.000153922	0.149633555	chr10	5454564
cg19564098	0.192191867	0.160990843	0.031201024	0.0001543	0.149633555	chr10	124895697
cg19811863	0.689577595	0.650304816	0.03927278	0.000154478	0.149633555	chr6	32785740
cg26909817	0.856706383	0.843389995	0.013316388	0.000155377	0.149781659	chr16	53218077
cg22888850	0.828799411	0.84479778	−0.015998369	0.000155619	0.149781659	chr13	53536706
cg24636244	0.634037866	0.604155345	0.02988252	0.000155913	0.149781659	chr10	6221185
cg13587495	0.805743938	0.790993702	0.014750236	0.000155944	0.149781659	chr3	89449406
cg09597390	0.683269913	0.649321859	0.033948054	0.000156663	0.15015561	chr11	7436013
cg22025570	0.897635118	0.906022876	−0.008387758	0.00015736	0.150179657	chr1	4832352
cg20818191	0.838732359	0.818317993	0.020414366	0.000157362	0.150179657	chr4	866154
cg23463205	0.733030488	0.749422613	−0.016392125	0.000157676	0.150179657	chr10	118305444
cg13386839	0.90556905	0.891733264	0.013835786	0.000158622	0.150766307	chr1	12250343
cg10945855	0.312751417	0.365151347	−0.05239993	0.00015941	0.151200621	chr7	63085215
cg17734983	0.5824649	0.598298298	−0.015833398	0.000159796	0.151251769	chr15	25456897
cg21884905	0.117271823	0.131580712	−0.014308889	0.000160375	0.151485845	chr8	144511672
cg23191740	0.765612353	0.726225346	0.039387007	0.000160821	0.151592741	chr21	46694944
cg22293261	0.929911373	0.916676505	0.013234868	0.00016296	0.152765778	chr17	79958141
cg14480943	0.762303882	0.741178481	0.021125401	0.000162995	0.152765778	chr3	20589760
cg06828282	0.857167122	0.84037031	0.016796813	0.000163141	0.152765778	chr11	47151756
cg20133000	0.094741519	0.107555837	−0.012814317	0.000163575	0.152765778	chr6	28219778
cg18617958	0.683289116	0.706833365	−0.023544248	0.000163739	0.152765778	chr2	192560369
cg26392005	0.092113594	0.083055369	0.009058225	0.000164415	0.153083405	chr14	81421297
cg09900527	0.858250309	0.871373036	−0.013122726	0.000166067	0.15430649	chr5	179859512
cg17269581	0.716038515	0.734116767	−0.018078252	0.000166418	0.154318762	chr17	16876375
cg11741432	0.641686326	0.604410007	0.037276319	0.000167929	0.155403626	chr17	4696249
cg02007235	0.614042866	0.629691762	−0.015648897	0.00016893	0.156013677	chr6	30568139
cg11185653	0.169855693	0.192996794	−0.023141102	0.000170288	0.156496769	chr14	105669480
cg09907183	0.774652725	0.791565396	−0.016912671	0.000170428	0.156496769	chr5	7273025
cg03334425	0.202852111	0.188094273	0.014757839	0.000170482	0.156496769	chr16	21675455
cg13608034	0.90706591	0.890100317	0.016965593	0.000172025	0.157595996	chr9	134762160
cg26406407	0.765865887	0.747635784	0.018230103	0.000173664	0.15846878	chr10	438229
cg22048274	0.681178848	0.657893341	0.023285508	0.000173673	0.15846878	chr6	31853428
cg13262159	0.879620757	0.88537587	−0.005755113	0.000174962	0.159326972	chr11	71230303
cg10814135	0.222268675	0.203209375	0.0190593	0.000176043	0.159671598	chr17	79935032
cg08460717	0.910332156	0.897755743	0.012576413	0.000176057	0.159671598	chr6	33149789
cg01976034	0.784767593	0.808024864	−0.023257272	0.000176562	0.159671598	chr3	57313955
cg25818915	0.732616599	0.744241549	−0.01162495	0.000176741	0.159671598	chrX	110863969
cg11537946	0.194281698	0.216167417	−0.021885719	0.000177447	0.159992959	chr4	139773369
cg18664773	0.088610544	0.076220744	0.0123898	0.000178154	0.160030382	chr19	59085049
cg07437360	0.59865481	0.629449061	−0.030794251	0.00017819	0.160030382	chr11	130413263
cg25719374	0.554195797	0.532995392	0.021200405	0.000178893	0.1602277	chr19	3930244
cg25420132	0.473661644	0.509697134	−0.03603549	0.000179203	0.1602277	chr6	21762391
cg06989693	0.644647691	0.669253756	−0.024606065	0.000179669	0.1602277	chr5	41409354
cg26429022	0.578434859	0.527063127	0.051371732	0.000179815	0.1602277	chr7	5186528
cg27212541	0.366319522	0.411987426	−0.045667905	0.000180331	0.160374261	chr3	49507385
cg23259384	0.856404913	0.840453381	0.015951532	0.000182144	0.161419482	chr3	14545312
cg16533336	0.769445535	0.723094279	0.046351256	0.000182214	0.161419482	chr7	70765583

cg20010054	0.685581447	0.703206844	−0.017625398	0.000186646	0.165025405	chr14	102714291
cg20211088	0.16862742	0.196966863	−0.028339442	0.000187061	0.165050633	chr6	13712555
cg04282497	0.691998302	0.708192867	−0.016194565	0.00018759	0.165050633	chr4	82350883
cg04628413	0.059131847	0.069015413	−0.009883566	0.00018776	0.165050633	chr16	28962222
cg12794168	0.852130882	0.862229016	−0.010098134	0.000189341	0.166120979	chr15	37110614
cg05825459	0.586572123	0.613904782	−0.027332659	0.000189961	0.166344334	chr2	205543455
cg03875082	0.816373274	0.833772487	−0.017399213	0.000190659	0.16663644	chr16	18483197
cg01122304	0.088995459	0.105783816	−0.016788357	0.000191666	0.167195677	chr4	2845576
cg19468946	0.888606748	0.868741342	0.019865406	0.000194217	0.169097689	chr17	37922297
cg05544828	0.926284212	0.917370713	0.008913499	0.000194793	0.169275951	chr6	35395209
cg10923702	0.900925355	0.907701278	−0.006775923	0.000195826	0.169850488	chr13	44542862
cg26940109	0.789661542	0.810630292	−0.02096875	0.000196207	0.169857686	chr1	32095849
cg14063468	0.875755744	0.852990276	0.022765467	0.00019756	0.170530056	chr9	123339570
cg25739700	0.72118634	0.697718089	0.02346825	0.000197731	0.170530056	chr17	5393371
cg02501155	0.937343805	0.927500909	0.009842896	0.000198148	0.170567417	chr16	79263473
cg01223171	0.882562179	0.866766038	0.01579614	0.000198935	0.170922472	chr4	61660358
cg09399236	0.12629288	0.139854903	−0.013562024	0.000200292	0.171002276	chr3	101293650
cg13943052	0.16113556	0.176851411	−0.015715852	0.000200426	0.171002276	chr15	40987167
cg03537567	0.658619438	0.642522982	0.016096456	0.000201135	0.171002276	chr4	25375829
cg24421865	0.833222808	0.811093734	0.022129074	0.000201298	0.171002276	chr21	45919623
cg07891737	0.68080739	0.699823145	−0.019015755	0.000201586	0.171002276	chr19	54215577
cg10726312	0.773020957	0.739962032	0.033058926	0.000201906	0.171002276	chr2	85837083
cg11402396	0.566351443	0.589087206	−0.022735763	0.000201932	0.171002276	chr10	13364856
cg19505364	0.780369988	0.795530564	−0.015160576	0.000202292	0.171002276	chr4	583022
cg07221511	0.945337992	0.937264145	0.008073847	0.000202401	0.171002276	chr3	164009569
cg15118745	0.675829451	0.696538272	−0.020708821	0.000205031	0.172903732	chr17	33580897
cg10476980	0.762228487	0.733070807	0.02915768	0.000207027	0.174264882	chrX	134051922
cg19733894	0.812361731	0.822614076	−0.010252345	0.000207605	0.174375036	chr2	33111822
cg13724788	0.253650125	0.220755886	0.03289424	0.000207922	0.174375036	chr19	19322778
cg14131834	0.566486998	0.590263215	−0.023776217	0.000208587	0.174611652	chr13	45914250
cg04524239	0.851632627	0.869482047	−0.01784942	0.000209963	0.175441732	chr15	25320886
cg11280668	0.680925581	0.657714847	0.023210735	0.00021128	0.175983458	chrX	17754811
cg11733626	0.144011978	0.15809598	−0.014084002	0.000211742	0.175983458	chr3	123304043
cg00643086	0.126237234	0.141466143	−0.015228909	0.000211769	0.175983458	chr6	26332252
cg19157724	0.864356396	0.852485366	0.01187103	0.000214072	0.176667885	chr2	98337094
ch.2.42814419F	0.224132804	0.238497668	−0.014364865	0.000214088	0.176667885	chr2	42960915
cg25204764	0.846995625	0.826017743	0.020977882	0.000214358	0.176667885	chr1	24995933
cg15088324	0.743137911	0.76367292	−0.020535009	0.000214747	0.176667885	chr19	6707621
cg13460740	0.827585245	0.841694393	−0.014109148	0.000215258	0.176667885	chr22	21369777
cg06112727	0.034941805	0.026725454	0.00821635	0.000215262	0.176667885	chr8	22102711
cg18834652	0.615811908	0.60083154	0.014980369	0.000215874	0.176667885	chr11	94276072
cg02039485	0.844219095	0.830784779	0.013434316	0.000216439	0.176667885	chr14	93570854
cg15061682	0.835359413	0.850471803	−0.01511239	0.000216644	0.176667885	chr13	102850387
cg06138195	0.615382819	0.638400389	−0.02301757	0.000216767	0.176667885	chr17	45143871
cg15751667	0.617908126	0.58367883	0.034229296	0.000216852	0.176667885	chr19	33574995
cg15936935	0.388783011	0.408473022	−0.019690011	0.000218294	0.177525332	chr14	105827276
cg11224984	0.911792337	0.90505005	0.006742287	0.000219336	0.177782078	chr1	2203045

cg24425342	0.952058655	0.946201457	0.005857197	0.000219447	0.177782078	chr19	3114929
cg20149362	0.642896233	0.607455784	0.035440449	0.000219886	0.177782078	chr1	7834809
cg13367929	0.965321277	0.959293615	0.006027661	0.000220269	0.177782078	chr5	176077110
cg14780883	0.66136414	0.676720914	−0.015356774	0.000220573	0.177782078	chr15	25466402
cg23912186	0.884833058	0.899900125	−0.015067066	0.000221176	0.177782078	chr1	207144812
cg05728519	0.083612166	0.102650016	−0.01903785	0.000221337	0.177782078	chr12	48551378
cg01764341	0.872205715	0.857040091	0.015165624	0.000222347	0.178107297	chr1	145095823
cg19901994	0.945234809	0.936414306	0.008820503	0.000222523	0.178107297	chr8	27288146
cg10698327	0.11917989	0.090722275	0.028457615	0.00022375	0.178316595	chr4	9534651
cg11522201	0.096244348	0.106835274	−0.010590926	0.000223886	0.178316595	chr9	95432733
cg20326359	0.819984024	0.83273962	−0.012755596	0.000223957	0.178316595	chr14	101450218
cg09773897	0.881549007	0.863499888	0.018049118	0.000224938	0.178785823	chr19	3364892
cg23028721	0.626685431	0.654385367	−0.027699937	0.000225661	0.178845534	chr1	248365229
cg03010018	0.902666647	0.892939996	0.009726652	0.000225797	0.178845534	chr11	116708299
cg14968543	0.264175447	0.294853752	−0.030678305	0.000226853	0.179081846	chr8	99986964
cg12785139	0.723634031	0.754592517	−0.030958486	0.00022688	0.179081846	chr2	130766434
cg08770523	0.085143751	0.101981701	−0.016837951	0.000227394	0.179177244	chr4	103266227
cg06080712	0.542845794	0.564492059	−0.021646264	0.000228409	0.179578219	chr22	32435515
cg13567025	0.088487598	0.095612001	−0.007124403	0.00022869	0.179578219	chr1	236958589
cg16058880	0.099366981	0.105885887	−0.006518906	0.000231169	0.180771684	chr1	145039638
cg01813209	0.448190837	0.468848913	−0.020658075	0.000231644	0.180771684	chr2	71615655
cg08066535	0.681874325	0.697967375	−0.01609305	0.000231685	0.180771684	chr6	11649444
cg21017641	0.918126631	0.928136194	−0.010009563	0.000231795	0.180771684	chr13	114786850
cg21229103	0.845602966	0.820696077	0.024906889	0.000232464	0.180888956	chr9	140738145
cg19678730	0.83351886	0.84769806	−0.0141792	0.000232738	0.180888956	chr12	9280567
cg18058682	0.800738106	0.818086771	−0.017348665	0.000233514	0.181121648	chr15	25466114
cg25227755	0.832164749	0.855559663	−0.023394914	0.000234316	0.181121648	chr20	37374470
cg00565075	0.252183646	0.297432944	−0.045249298	0.000234368	0.181121648	chr22	38244893
cg27574244	0.173418827	0.189285476	−0.015866649	0.000234626	0.181121648	chr17	80709357
cg09502339	0.884595671	0.870454106	0.014141565	0.00023575	0.181682507	chr6	33152642
cg00936904	0.713130511	0.776337744	−0.063207233	0.000236177	0.181704134	chr4	190731885
cg01127291	0.732873396	0.700690261	0.032183136	0.000237115	0.181824628	chrX	63424405
cg01745539	0.729652936	0.657496722	0.072156214	0.00023713	0.181824628	chr6	32632331
cg27527798	0.741694224	0.709251441	0.032442783	0.000238714	0.182494294	chr7	1773245
cg22991512	0.20464014	0.185019356	0.019620784	0.000238804	0.182494294	chr3	14643854
cg08836619	0.227917412	0.250354651	−0.022437239	0.000239651	0.182561514	chr5	139555389
cg01541565	0.615477529	0.570604893	0.044872637	0.000239692	0.182561514	chr6	32606385
cg10532407	0.863875311	0.879512906	−0.015637595	0.000240263	0.182691034	chr3	21448012
cg18761118	0.742518736	0.72660581	0.015912926	0.000241903	0.183344674	chr1	53388030
cg01858764	0.851303843	0.83601267	0.015291172	0.000241926	0.183344674	chr11	118862530
cg10568796	0.724891972	0.69804283	0.026849142	0.000243911	0.184413704	chr22	21193857
cg06060754	0.646747501	0.479560634	0.167186867	0.000244145	0.184413704	chr5	176797920
cg14066075	0.916272245	0.905108088	0.011164157	0.000245771	0.184636551	chr9	140325583
cg26624273	0.097037459	0.106314252	−0.009276793	0.00024628	0.184636551	chr1	38019894
cg21848624	0.854812672	0.764123788	0.090688883	0.000246315	0.184636551	chr18	77219388
cg08273502	0.764043558	0.776669355	−0.012625797	0.000246592	0.184636551	chr14	84979439
cg01896432	0.738965387	0.765305381	−0.026339994	0.000246724	0.184636551	chr4	68904160

cg03298314	0.72845052	0.691624636	0.036825884	0.000246868	0.184636551	chr6	30311668
cg27513204	0.797844131	0.810204975	−0.012360843	0.000247998	0.184661377	chr1	222673618
cg24793265	0.888910795	0.876272327	0.012638468	0.000248114	0.184661377	chr9	139269240
cg01315787	0.891978904	0.872949556	0.019029348	0.000248116	0.184661377	chr5	173450571
cg09563846	0.962720758	0.96746885	−0.004748092	0.000248929	0.18496473	chr11	875758
cg06947694	0.916848593	0.90747454	0.009374053	0.000250148	0.185305568	chr7	5389271
cg21158502	0.404877695	0.361716035	0.043161661	0.000250212	0.185305568	chr5	74348187
cg27558479	0.098405277	0.107642977	−0.0092377	0.00025067	0.185305568	chr2	74425610
cg25642880	0.819550057	0.831666605	−0.012116548	0.000251012	0.185305568	chr13	96581176
cg01412011	0.770493128	0.748163427	0.022329701	0.000251758	0.185555723	chr4	3652433
cg23981611	0.822191773	0.791908828	0.030282945	0.000252794	0.18585813	chr6	30167399
cg22455795	0.427841406	0.457989767	−0.030148361	0.000253286	0.18585813	chr19	41109651
cg27399012	0.069418573	0.057446519	0.011972054	0.00025339	0.18585813	chr17	16342849
cg13284614	0.925890848	0.920122083	0.005768765	0.000253856	0.185900722	chr3	52569169
cg03983398	0.864657577	0.854331059	0.010326518	0.000254276	0.185909901	chr1	45254530
cg25754733	0.931960824	0.926450214	0.00551061	0.00025618	0.187002593	chr19	640965
cg02052377	0.65048077	0.623219069	0.027261701	0.000257894	0.187952679	chr11	10674182
cg23009962	0.915711339	0.911397401	0.004313938	0.000258434	0.188046274	chr19	18572435
cg13003239	0.516506829	0.535248899	−0.018742071	0.000259252	0.188340769	chr3	49044627
cg02542817	0.907885162	0.92446654	−0.016581378	0.000260584	0.189007939	chr2	229291442
cg23762263	0.433944395	0.393793711	0.040150684	0.000261655	0.189350708	chr2	154730326
cg15412228	0.963238671	0.956281535	0.006957136	0.000261887	0.189350708	chr14	104718313
cg08782677	0.786566286	0.762482254	0.024084032	0.000262555	0.189533552	chrX	150346292
cg09242008	0.929163408	0.91980694	0.009356468	0.000263544	0.189897182	chr3	108839099
cg26657101	0.820041075	0.833593126	−0.013552051	0.000264163	0.189897182	chr15	97322869
cg07280582	0.788121942	0.81203362	−0.023911679	0.000264542	0.189897182	chr12	53854172
cg16336120	0.312677213	0.287262466	0.025414747	0.000264724	0.189897182	chr7	45197318
cg25059445	0.732824184	0.750534643	−0.017710459	0.000265304	0.190014768	chr4	178366587
cg03860054	0.46545222	0.485468853	−0.020016633	0.000266329	0.190449271	chr16	55691102
cg02327123	0.13471752	0.123466451	0.011251068	0.000267662	0.190548842	chr7	101006363
cg11716064	0.45511685	0.479564047	−0.024447197	0.000267872	0.190548842	chr10	129477313
cg17509989	0.591391829	0.393038177	0.198353652	0.000268109	0.190548842	chr5	176798049
cg22550003	0.938700665	0.930362538	0.008338127	0.000268873	0.190548842	chr15	44170550
cg06684407	0.863435187	0.842468971	0.020966215	0.00026896	0.190548842	chr21	46356806
cg08888487	0.886793585	0.895968679	−0.009175094	0.000269312	0.190548842	chr5	1124759
cg05744487	0.541050741	0.513661332	0.027389409	0.000269804	0.190548842	chr11	18720220
cg19081571	0.924705285	0.916753794	0.007951492	0.000269809	0.190548842	chr1	1190137
cg01794265	0.083898176	0.092751278	−0.008853102	0.000272201	0.191755714	chr5	14582549
cg18391747	0.619161065	0.646424972	−0.027263907	0.000272584	0.191755714	chr1	161375390
cg06151505	0.715577558	0.731757869	−0.01618031	0.000273156	0.191755714	chr3	138866874
cg24865495	0.917582358	0.907307351	0.010275008	0.000273963	0.191755714	chr11	63912769
cg22016770	0.668688906	0.690252591	−0.021563685	0.000274219	0.191755714	chr5	42468779
cg11675409	0.747634541	0.730290224	0.017344317	0.000274243	0.191755714	chr15	44083712
cg24152845	0.608324883	0.628187058	−0.019862175	0.000274865	0.191755714	chr15	25414763
cg06253862	0.706505764	0.735740359	−0.029234595	0.00027488	0.191755714	chr6	160201362
cg03464692	0.815493678	0.835272362	−0.019778684	0.000277433	0.193240621	chr4	1631517
cg00649216	0.801719158	0.820013231	−0.018294073	0.000277884	0.193259958	chr6	63921501

cg08048222	0.185963749	0.207834817	−0.021871068	0.000278373	0.193305278	chr19	58239012
cg15393022	0.892906673	0.882760543	0.01014613	0.000280241	0.194306872	chr1	54775837
cg19892525	0.469084954	0.493428895	−0.02434394	0.000281138	0.194484369	chr11	108157498
cg13882665	0.742648091	0.757076928	−0.014428837	0.000281698	0.194484369	chr6	169560079
cg15825373	0.750666845	0.725543259	0.025123586	0.000281815	0.194484369	chr1	947153
cg01575164	0.714362906	0.729474296	−0.01511139	0.000282202	0.194484369	chr3	197515016
cg12679725	0.942994866	0.954417633	−0.011422767	0.000284382	0.195606122	chr22	42551834
cg09412808	0.860331424	0.852670522	0.007660903	0.000284987	0.195606122	chr4	57180590
cg06251958	0.598259794	0.615495354	−0.01723556	0.000285116	0.195606122	chr7	102989081
cg27466615	0.924594677	0.91339647	0.011198207	0.00028699	0.195965977	chr17	80678315
cg24621371	0.765118695	0.744348292	0.020770404	0.000287086	0.195965977	chr12	125531715
cg00559790	0.088428407	0.099099962	−0.010671555	0.000287119	0.195965977	chr1	93914052
cg02592416	0.867907418	0.878602019	−0.0106946	0.000287922	0.195965977	chr12	52680161
cg08866814	0.94489444	0.936581538	0.008312902	0.000288111	0.195965977	chr16	89351500
cg05735180	0.607043895	0.564470802	0.042573093	0.000288218	0.195965977	chr18	55095177
cg09622269	0.588894194	0.622904268	−0.034010075	0.00028948	0.196285281	chr10	76803925
cg15019005	0.722928867	0.737784067	−0.0148552	0.00028978	0.196285281	chr5	7825313
cg13898697	0.913325257	0.908460482	0.004864775	0.000289978	0.196285281	chr1	36641773
cg08742360	0.817221173	0.804605253	0.01261592	0.000291031	0.196706091	chr6	149019254
cg13534503	0.958462717	0.964821733	−0.006359016	0.0002917	0.196866535	chr9	139535765
cg26473110	0.76182225	0.742990695	0.018831555	0.000293379	0.197607128	chr11	46385365
cg14145438	0.927958625	0.916704929	0.011253696	0.000293699	0.197607128	chr3	43428668
cg10169812	0.907399315	0.891423454	0.015975861	0.000294097	0.197607128	chr4	15779392
cg17897352	0.876081556	0.885908908	−0.009827352	0.00029529	0.197752564	chr10	121011644
cg23530263	0.234654116	0.264149869	−0.029495753	0.000295452	0.197752564	chr19	55996431
cg10741826	0.919710192	0.915910812	0.00379938	0.00029685	0.197752564	chr11	128680466
cg13152990	0.856647504	0.877000852	−0.020353349	0.000297024	0.197752564	chr13	39584965
cg11075121	0.107117969	0.12023474	−0.013116771	0.000297085	0.197752564	chr12	120967065
cg07141527	0.741062131	0.717492485	0.023569645	0.000297332	0.197752564	chr12	121202554
cg17079378	0.023224868	0.03044584	−0.007220972	0.000297458	0.197752564	chr1	46713440
cg06186457	0.398790194	0.417121632	−0.018331438	0.000298186	0.197752564	chr6	4504273
cg11601443	0.857664303	0.839656341	0.018007962	0.000298214	0.197752564	chr12	113415930
cg14700153	0.937392538	0.930579922	0.006812615	0.000300067	0.198271306	chr18	77515904
cg23197881	0.113501352	0.128224005	−0.014722653	0.000300249	0.198271306	chr12	70133421
cg26353844	0.495879766	0.521342055	−0.02546229	0.00030064	0.198271306	chr1	95133907
cg23530053	0.122324786	0.136151756	−0.01382697	0.000301504	0.198271306	chr18	20513673
cg22529861	0.111732076	0.120395667	−0.008663591	0.000301543	0.198271306	chr11	11643697
cg04830812	0.579047891	0.548687897	0.030359995	0.000301604	0.198271306	chr12	7074724
cg05086567	0.901240296	0.907659327	−0.006419032	0.000303085	0.198958127	chr17	7486615
cg08731817	0.864353941	0.845380334	0.018973607	0.000305014	0.199349794	chr6	158443177
cg23465426	0.445005759	0.461877079	−0.016871321	0.000305338	0.199349794	chr6	32847377
cg04111029	0.779955779	0.753588012	0.026367767	0.000305614	0.199349794	chr2	17718449
cg07578410	0.157799191	0.178531155	−0.020731964	0.000305866	0.199349794	chr5	171710114

Supplementary Table S2: CpGs associated with E2 levels in PCOS patients

Row.names	intercept	beta	t	pval	qval	chr	pos
cg23363602	0.102166234	0.000817118	8.513428889	2.96E–09	0.000824783	chr6	29933971
cg11136592	0.984405633	−0.001792114	−8.454523353	3.41E–09	0.000824783	chr10	5246625
cg13551074	0.925127162	−0.001251204	−7.548275307	3.19E–08	0.003682075	chr2	243017952
cg00836091	0.120749179	−0.000270603	−7.433627511	4.27E–08	0.003682075	chr3	53290192
cg02031121	1.042458795	−0.001966545	−7.407831518	4.56E–08	0.003682075	chr15	52331471
cg15027633	1.009287773	−0.001623296	−7.407381353	4.57E–08	0.003682075	chr6	14771388
cg08872590	0.010982342	0.000707927	7.25610341	6.72E–08	0.004027238	chr8	145748059
cg26537272	0.987450513	−0.001543158	−7.238354418	7.03E–08	0.004027238	chr5	1214606
cg11005027	1.006946104	−0.002397732	−7.207541655	7.61E–08	0.004027238	chr11	134237890
cg24983605	0.946537249	−0.001372675	−7.138973695	9.08E–08	0.004027238	chr2	180487919
cg16114706	0.91656486	−0.001117874	−7.135666408	9.16E–08	0.004027238	chr22	46509464
cg01726982	0.965072066	−0.001348006	−6.877137527	1.79E–07	0.007214339	chr1	228630108
cg18266400	1.039032765	−0.002090381	−6.802361278	2.18E–07	0.007547297	chr3	124990403
cg05146395	0.975735319	−0.001888482	−6.722094178	2.68E–07	0.007547297	chr20	61314143
cg03884018	1.055553325	−0.002715018	−6.708728487	2.78E–07	0.007547297	chr16	26044240
cg18988159	0.998456927	−0.001776554	−6.695506883	2.88E–07	0.007547297	chr8	33459821
cg12578563	1.039350345	−0.001836262	−6.681426845	2.99E–07	0.007547297	chr11	1159210
cg08466517	1.095352704	−0.003463201	−6.670940596	3.07E–07	0.007547297	chr4	122138632
cg15423862	0.977504675	−0.00093381	−6.66973127	3.08E–07	0.007547297	chr3	155838109
cg07560948	0.885211851	−0.001145255	−6.662018199	3.14E–07	0.007547297	chr1	41264498
cg26008260	1.005072573	−0.001433416	−6.638965971	3.34E–07	0.007547297	chr2	25057298
cg23934075	0.058674222	0.000644621	6.628537809	3.43E–07	0.007547297	chr6	29933697
cg06686422	0.995510621	−0.002174586	−6.604626698	3.66E–07	0.007688472	chr5	174174566
cg07719523	0.986737203	−0.001711395	−6.577992408	3.92E–07	0.007904158	chr19	746526
cg07370274	0.9713068	−0.001350294	−6.560045298	4.11E–07	0.007956019	chr9	29825210
cg18098534	0.97871495	−0.001429284	−6.538160023	4.36E–07	0.007967486	chr14	86088124
cg19453472	0.982383986	−0.002095055	−6.530363221	4.45E–07	0.007967486	chr7	1139076
cg04842352	0.101764429	0.00218088	6.513497387	4.65E–07	0.008033253	chr16	49907368
cg20006652	0.792518388	−0.001261566	−6.49618871	4.87E–07	0.008094732	chr6	142238173
cg07592775	1.010080904	−0.002513548	−6.484541406	5.02E–07	0.008094732	chr1	19210017
cg09730955	0.982416577	−0.001491325	−6.444471677	5.58E–07	0.008710969	chr5	143130392
cg04644773	0.098265839	0.000591849	6.424436701	5.89E–07	0.008899302	chr5	179636283
cg16919579	0.806435661	−0.00106288	−6.402249685	6.24E–07	0.009153098	chr2	157632795
cg18523346	0.950634468	−0.001349508	−6.382363322	6.58E–07	0.009365792	chr4	1729959
cg19536902	1.049122177	−0.002368287	−6.302565732	8.14E–07	0.011058543	chr11	13305152
cg09259797	0.993810453	−0.001350703	−6.298453801	8.23E–07	0.011058543	chr13	106650225
cg02038418	1.022326208	−0.001367376	−6.281969319	8.60E–07	0.011243031	chr15	39958882
cg11932575	0.956522474	−0.001274017	−6.26888896	8.90E–07	0.011291198	chr4	128048177
cg25165144	0.938144242	−0.001937173	−6.260631399	9.10E–07	0.011291198	chr12	670974
cg09730302	0.939598714	−0.002150691	−6.23620939	9.72E–07	0.011750563	chr10	128595047
cg07784793	1.009773231	−0.001626202	−6.223817666	1.00E–06	0.011849775	chr5	33794720
cg16437009	0.051013097	0.002883909	6.208226652	1.05E–06	0.012059789	chr10	98945799
cg23598886	0.902376237	−0.000717029	−6.192401042	1.09E–06	0.012077031	chr18	12777645
cg08026818	0.080613733	0.000238686	6.181893102	1.12E–06	0.012077031	chr16	77823221
cg09532664	0.993697121	−0.002073933	−6.183425746	1.12E–06	0.012077031	chr5	9631216

cg26197930	1.000250414	−0.002710581	−6.1661697	1.17E–06	0.012322202	chr5	1222775
cg00807237	0.930401632	−0.001287438	−6.156806948	1.20E–06	0.012366131	chr7	934156
cg11176525	0.968836611	−0.001038188	−6.113353515	1.35E–06	0.013603585	chr3	45003829
cg05483487	0.90063237	−0.002092788	−6.088966746	1.44E–06	0.013736558	chr12	670892
cg08836825	0.91831665	−0.001138895	−6.087847778	1.45E–06	0.013736558	chr6	82726757
cg07836887	0.970957939	−0.000959096	−6.077207083	1.49E–06	0.013736558	chr17	5346448
cg00048705	0.975200399	−0.001194602	−6.066490357	1.53E–06	0.013736558	chr6	170563326
cg05798111	0.960355763	−0.001707681	−6.063900289	1.54E–06	0.013736558	chr16	68560845
cg04024413	0.045709707	0.000497755	6.05960211	1.56E–06	0.013736558	chr6	27782541
cg21630370	0.88423895	−0.001249349	−6.05899613	1.56E–06	0.013736558	chr7	45141979
cg03570577	0.880979856	−0.000940478	−6.04137466	1.64E–06	0.014042959	chr6	29765324
cg08320316	0.977598548	−0.001095686	−6.037488313	1.65E–06	0.014042959	chr7	5812747
cg03085859	0.605584326	−0.000378136	−5.965093201	2.01E–06	0.016768067	chr6	29457094
cg27509366	0.966084078	−0.001497949	−5.949699299	2.10E–06	0.017155048	chr8	142149222
cg26977256	0.906259875	−0.000514391	−5.944036768	2.13E–06	0.017155048	chr6	32022470
cg05780294	0.943688775	−0.001348351	−5.929062056	2.22E–06	0.017568703	chr7	128312789
cg06880420	0.917687164	−0.001437508	−5.913685325	2.31E–06	0.018016985	chr1	200119987
cg16771467	0.939395563	0.000132464	5.871948166	2.58E–06	0.019602154	chr18	55315872
cg02747151	1.015507363	−0.001304901	−5.870574733	2.59E–06	0.019602154	chr15	25981177
cg22829451	0.948169278	−0.000946094	−5.864923058	2.63E–06	0.019602154	chrX	70151816
cg17822978	0.979140673	−0.001112134	−5.850586092	2.74E–06	0.020067263	chr15	72073485
cg13085030	1.006686713	−0.002093239	−5.829056711	2.90E–06	0.020951678	chr7	1090504
cg24709001	0.979009132	−0.001017204	−5.82357548	2.95E–06	0.020951678	chr4	3235319
cg10541332	0.087687504	0.000767506	5.816372806	3.00E–06	0.021053982	chr3	61237712
cg07830126	0.923727736	−0.001560464	−5.804234672	3.10E–06	0.02128189	chr1	3477690
cg07341073	0.920425244	0.000283371	5.801823128	3.12E–06	0.02128189	chr2	175300689
cg07170252	0.985493171	−0.001778107	−5.772753421	3.38E–06	0.022525126	chr14	76870544
cg26996890	0.974780924	−0.001335019	−5.770568583	3.40E–06	0.022525126	chr7	1980808
cg19205926	0.88259869	−0.000730957	−5.757432212	3.52E–06	0.023025056	chr4	6757569
cg01758856	0.93419929	−0.000348531	−5.708830688	4.02E–06	0.025914615	chr17	80814611
cg19987253	0.884869383	−0.000440388	−5.700130628	4.11E–06	0.025971281	chr7	1981314
cg11004284	0.179800467	0.001736618	5.698315461	4.13E–06	0.025971281	chr3	15470105
cg01267068	0.937999507	−0.001341237	−5.633158125	4.93E–06	0.030595128	chr2	731158
cg04218124	0.86796176	−0.001119482	−5.600567351	5.39E–06	0.033003053	chr2	219284345
cg20675194	0.949390537	−0.000790818	−5.580788159	5.69E–06	0.034389735	chr11	58694219
cg01380321	0.593127204	−0.001794877	−5.523156076	6.65E–06	0.039556297	chr15	98631681
cg11753155	0.094166023	0.001437609	5.51921389	6.72E–06	0.039556297	chr6	29760148
cg09790780	0.868882545	−0.000279469	−5.511484849	6.87E–06	0.039556297	chr16	7482013
cg11419575	0.932036512	−0.001042904	−5.511368989	6.87E–06	0.039556297	chr2	153057378
cg07834476	0.046441822	0.000244673	5.505235676	6.98E–06	0.039748761	chr10	25305695
cg15714607	0.076449231	0.000839988	5.461041203	7.88E–06	0.044308765	chr11	75526013
cg00536383	0.065423596	0.000799665	5.439037952	8.36E–06	0.046504432	chr2	9346592
cg15861540	0.088759863	0.000275173	5.395163826	9.43E–06	0.051814471	chr19	3606463
cg24737193	0.897404385	−0.001018335	−5.382602384	9.75E–06	0.053016958	chr18	12778029
cg17924813	0.134474248	0.000475996	5.333877422	1.11E–05	0.059220325	chr4	15704844
ch.12.859106F	0.108930101	−0.000229314	−5.334056782	1.11E–05	0.059220325	chr12	42882094
cg00984474	0.214047937	−0.000465545	−5.314147629	1.18E–05	0.061816063	chr5	7850922

cg22466043	0.036028665	0.001443976	5.303692282	1.21E–05	0.062921129	chr10	38692145
cg21193744	0.933520485	−0.001203821	−5.290677739	1.25E–05	0.064502532	chr8	6405105
cg13168820	0.129993803	0.000340323	5.273340292	1.31E–05	0.066916496	chr20	41818356
cg11298989	0.022287439	0.000229387	5.257909472	1.37E–05	0.069068611	chr19	54960853
cg27121529	0.04777533	0.000287343	5.249666629	1.40E–05	0.069912414	chr7	99102251
cg01810111	0.098625023	0.000675412	5.217344121	1.53E–05	0.07558476	chr6	27637395
cg15946590	0.334291769	0.000973453	5.204088154	1.59E–05	0.077580004	chr6	30421089
cg07301433	0.183595193	−0.000552222	−5.192995488	1.64E–05	0.079167117	chr2	38303999
cg11226480	0.033235333	0.000366614	5.1884756	1.66E–05	0.079357135	chr12	51442072
cg17093826	0.834498367	0.000328261	5.183929628	1.68E–05	0.079561112	chr7	138458720
cg02599587	0.8867479	−0.000295687	−5.16708152	1.76E–05	0.082500237	chr5	153275285
cg09635053	0.058472989	0.000870174	5.115945361	2.02E–05	0.0939614	chr16	1031944
cg25921736	0.848271035	−0.000360112	−5.10790294	2.06E–05	0.09441414	chr7	139971436
cg12871376	0.728546023	−0.000810979	−5.107217768	2.07E–05	0.09441414	chr6	41716240
cg08506127	0.723385109	−0.000621008	−5.101908231	2.10E–05	0.094899089	chr2	87018958
cg00323861	0.961745364	−0.002405434	−5.063366856	2.33E–05	0.103577742	chr4	70155747
cg18339359	1.05530747	−0.003702409	−5.063122382	2.33E–05	0.103577742	chr8	23423757
cg20009461	0.93996791	0.000108164	5.053701962	2.39E–05	0.104365053	chr17	80560634
cg22272840	0.251290957	0.00111238	5.054962875	2.39E–05	0.104365053	chr8	10586882
cg04007531	0.093946093	0.001708624	5.041905524	2.47E–05	0.106823502	chr6	29933511
cg12601945	0.141282573	0.000598153	5.020752127	2.62E–05	0.112182347	chr1	18958924
cg18538138	0.915956922	0.000256249	5.013374924	2.67E–05	0.113464058	chr2	33604572
cg13934625	0.34369677	−0.000645956	−5.008161131	2.71E–05	0.114092373	chr15	52472770
cg06068179	0.981427178	−0.001610229	−4.99359832	2.82E–05	0.117703629	chr4	7814047
cg21816685	0.097115529	0.00041199	4.982199339	2.91E–05	0.120392253	chr8	2023317
cg11181587	0.832692382	−0.000427394	−4.947875743	3.20E–05	0.13012729	chr10	121772185
cg12254517	0.827643066	−0.001018672	−4.947563938	3.20E–05	0.13012729	chr12	93454991
cg11814464	0.116662591	0.000383607	4.910578418	3.54E–05	0.142776032	chr18	7567548
cg16364155	0.909163446	−0.000462254	−4.900578481	3.64E–05	0.143447304	chr3	147144980
cg25006249	0.908276343	−0.001340334	−4.901020371	3.64E–05	0.143447304	chr20	259898
cg03698374	0.778885228	−0.001608698	−4.899832223	3.65E–05	0.143447304	chr4	1362912
cg16769381	0.93313552	0.000137472	4.887209582	3.78E–05	0.147287077	chr11	46391053
cg13225413	0.163132016	0.000520892	4.881033621	3.84E–05	0.148596926	chr17	5015653
cg17529745	0.857542225	0.000187035	4.872982534	3.92E–05	0.150660718	chr10	131708358
cg09911982	0.142222525	−0.000292841	−4.870183088	3.96E–05	0.150660718	chr6	33216283
cg15908308	0.869143781	−0.000312865	−4.856617533	4.10E–05	0.155131816	chr4	43459582
cg23941963	0.863877849	0.000185353	4.847368198	4.21E–05	0.157871195	chr7	204401
cg10189774	0.42861117	−0.000587286	−4.839765531	4.30E–05	0.159946821	chr4	17578691
cg15504747	0.058694223	0.000557087	4.819578244	4.54E–05	0.167648751	chr3	129312979
cg05345996	0.886603825	−0.000423924	−4.816975988	4.57E–05	0.167648751	chr10	133508028
cg17214754	0.869726442	0.00024993	4.800389931	4.79E–05	0.174105002	chr11	3168993
cg11401278	0.816926258	0.000324495	4.794614033	4.86E–05	0.175320554	chr11	2140096
cg06398643	0.905602326	0.000224261	4.792383265	4.89E–05	0.175320554	chr3	187896120
cg24631102	0.858489572	−0.000438155	−4.766771088	5.25E–05	0.186648898	chr10	129349644
cg13885748	0.113837676	0.000308374	4.76062045	5.34E–05	0.188426869	chr9	112232127
cg02679503	0.840303571	−0.000404346	−4.757629012	5.38E–05	0.188596739	chr2	42794680
cg16092346	0.945388778	−0.001382178	−4.754224728	5.43E–05	0.188989705	chr4	1363885

cg08352786	0.820009568	−0.000661013	−4.748528717	5.52E−05	0.190582855	chr12	81100403
cg04831495	0.948532415	−0.000278099	−4.738353511	5.67E−05	0.194565546	chr15	85060580
cg11265952	0.766522791	0.00022881	4.732814918	5.76E−05	0.196140819	chr3	57231292
cg19216044	0.960500897	−0.000210865	−4.728137945	5.83E−05	0.197273639	chr1	155005184

Supplementary Table S3: CpGs associated with PRL levels in PCOS patients

Row.names	intercept	beta	t	pval	qval	chr	pos
cg23339709	0.923410434	−0.002325525	−11.25788401	6.61E−12	2.40E−06	chr11	56258485
cg01847062	0.98549583	−0.008133101	−11.02809638	1.06E−11	2.40E−06	chr17	79792936
cg12046183	0.914340973	−0.005740527	−10.7056511	2.09E−11	2.40E−06	chr6	29859829
cg08017858	0.006825524	0.00797058	10.69719858	2.13E−11	2.40E−06	chr11	74022642
cg00409917	-0.001181505	0.003318406	10.626565	2.47E−11	2.40E−06	chr6	29894679
cg01062395	0.018308832	0.006598113	10.51334641	3.15E−11	2.55E−06	chr6	29855636
cg26649688	0.963318606	−0.004027965	−10.31511746	4.83E−11	2.90E−06	chr6	29858360
cg21557672	0.881455124	−0.003180453	−10.31196644	4.86E−11	2.90E−06	chr19	34315334
cg23238734	0.975924954	−0.003366653	−10.24856415	5.58E−11	2.90E−06	chr17	78661607
cg06655494	0.958887841	−0.003041032	−10.20408913	6.15E−11	2.90E−06	chr7	5415468
cg08186575	0.971461468	−0.003710828	−10.17364661	6.57E−11	2.90E−06	chr19	4371730
cg18274896	0.976863355	−0.003649976	−10.09057804	7.88E−11	3.19E−06	chr9	140395754
cg05611414	0.936897663	−0.003520704	−10.01830302	9.24E−11	3.24E−06	chr19	50643613
cg18805486	0.924095193	−0.004306019	−9.986858685	9.91E−11	3.24E−06	chr8	12551033
cg02632583	0.98336161	−0.002955336	−9.953417083	1.07E−10	3.24E−06	chr17	79253990
cg12619747	0.96193994	−0.004958517	−9.937075597	1.11E−10	3.24E−06	chr8	55180538
cg26353469	0.067382582	0.003384913	9.925609531	1.13E−10	3.24E−06	chr6	29855970
cg21331461	0.117129758	0.004510007	9.889338665	1.23E−10	3.25E−06	chr12	21927773
cg24179288	0.961297687	−0.002654475	−9.85120255	1.34E−10	3.25E−06	chr6	29867285
cg06064954	0.949513077	−0.003583791	−9.819278914	1.44E−10	3.25E−06	chr6	29869838
cg07811074	0.965133353	−0.003864891	−9.812616556	1.46E−10	3.25E−06	chr2	98350847
cg16223220	0.013781763	0.006213929	9.808351141	1.47E−10	3.25E−06	chr6	29855914
cg08231349	0.047125872	0.004140812	9.758562068	1.65E−10	3.34E−06	chr6	29894644
cg18423635	0.997192502	−0.003684488	−9.757292344	1.65E−10	3.34E−06	chr6	29869936
cg00598600	0.940960254	−0.004606751	−9.64953085	2.10E−10	4.08E−06	chr8	12610812
cg23266747	0.925513915	−0.003088461	−9.591212846	2.40E−10	4.44E−06	chr14	73168082
cg12801256	0.922098866	−0.003206231	−9.578592628	2.47E−10	4.44E−06	chr2	107459860
cg26419287	0.93777886	−0.00375833	−9.529247029	2.76E−10	4.79E−06	chr14	52482697
cg01101677	0.951165698	−0.004005535	−9.502842029	2.93E−10	4.91E−06	chr2	44313980
cg05989746	0.96365687	−0.003310684	−9.406513381	3.65E−10	5.91E−06	chr2	3425584
cg07754829	0.976021219	−0.001817582	−9.336612325	4.28E−10	6.71E−06	chr8	12551103
cg10148293	0.875820951	−0.002475955	−9.316594586	4.48E−10	6.80E−06	chr10	37413676
cg07273125	0.096909656	0.002503676	9.301960627	4.64E−10	6.82E−06	chr16	68295692
cg01718254	0.186596246	0.003328913	9.266358049	5.03E−10	7.16E−06	chr14	103995324
cg14893857	0.075062411	0.001782347	9.255489279	5.16E−10	7.16E−06	chr14	102554969
cg20578893	0.947236454	−0.003112739	−9.212532593	5.70E−10	7.69E−06	chr6	29870060
cg04459091	0.957980519	−0.003459861	−9.185006838	6.07E−10	7.97E−06	chr5	179751794
cg11079354	0.110958023	0.003783977	9.16785279	6.32E−10	8.07E−06	chr6	27740361
cg21549632	0.062537574	0.004656969	9.133801089	6.84E−10	8.51E−06	chr6	29856287
cg05074385	0.948113548	−0.002660126	−9.122567923	7.02E−10	8.52E−06	chr6	30137208

cg00453190	0.963315174	−0.00490928	−9.107026891	7.28E–10	8.62E–06	chr7	149460972
cg17865718	0.917809725	−0.003111162	−9.032321679	8.66E–10	9.92E–06	chr3	103926553
cg16555341	0.081027951	0.001489833	9.021489138	8.88E–10	9.92E–06	chr16	89160798
cg14230280	0.900521617	−0.002840325	−9.015612816	9.00E–10	9.92E–06	chr9	132502800
cg14667780	0.95433935	−0.002894131	−9.006714755	9.19E–10	9.92E–06	chr2	174613232
cg02223784	0.861663473	−0.002609368	−8.993345632	9.49E–10	1.00E–05	chr7	128589932
cg07983869	0.909674197	−0.002752721	−8.960044355	1.03E–09	1.05E–05	chr19	17802633
cg10474018	0.958955118	−0.002496225	−8.955458875	1.04E–09	1.05E–05	chr6	29860016
cg03177972	0.98901795	−0.003025034	−8.944923924	1.06E–09	1.05E–05	chr11	78480035
cg01793445	0.87412433	−0.003118531	−8.907628638	1.16E–09	1.11E–05	chr1	167632415
cg22354618	0.96242222	−0.002588483	−8.906080842	1.16E–09	1.11E–05	chr18	8800458
cg01723291	0.075296682	0.004346303	8.894500799	1.20E–09	1.12E–05	chr6	29855890
cg00943269	0.905953439	−0.001504248	−8.819466163	1.43E–09	1.31E–05	chr8	12550523
cg18325044	0.955198606	−0.002049413	−8.77151514	1.60E–09	1.44E–05	chr6	29868900
cg01876435	0.895887313	−0.002671208	−8.756739164	1.66E–09	1.46E–05	chr8	12545609
cg02227034	0.95537738	−0.002757632	−8.711178691	1.84E–09	1.59E–05	chr10	128080679
cg14973360	0.965421987	−0.004077322	−8.705077519	1.87E–09	1.59E–05	chr2	9800511
cg01398709	0.86769668	−0.004488268	−8.629827287	2.24E–09	1.87E–05	chr3	13918750
cg03593259	0.078320123	0.002680631	8.55950044	2.65E–09	2.18E–05	chr11	63321562
cg13946128	0.919691188	−0.004696372	−8.552174058	2.70E–09	2.18E–05	chr1	52372768
cg20537760	0.88579465	−0.003220536	−8.508360043	3.00E–09	2.38E–05	chr18	77534274
cg24528297	0.055884572	0.001646642	8.500500674	3.05E–09	2.39E–05	chr14	103995361
cg04939944	0.981243533	−0.00364283	−8.482802363	3.19E–09	2.45E–05	chr7	5390509
cg07831312	0.921982722	−0.003525331	−8.396419989	3.92E–09	2.98E–05	chr7	30855293
cg03995122	0.027878421	0.002281879	8.340062888	4.50E–09	3.36E–05	chr6	29894642
cg22945457	0.783841266	−0.002782118	−8.311040983	4.82E–09	3.55E–05	chr1	225612608
cg14117565	0.996834853	−0.009102028	−8.304689293	4.90E–09	3.55E–05	chr19	51850506
cg26127187	0.941395894	−0.003638573	−8.257299416	5.50E–09	3.93E–05	chr6	29856795
cg02687592	0.848986376	−0.003093388	−8.06838964	8.73E–09	6.14E–05	chr22	44708843
cg14667175	0.849444855	−0.002717311	−8.020032681	9.84E–09	6.81E–05	chr2	111623300
cg02523305	0.917403021	−0.003294413	−8.015105902	9.96E–09	6.81E–05	chr3	33263532
cg08545169	0.907243528	−0.001405803	−7.997647829	1.04E–08	7.01E–05	chr1	161169143
cg19617213	0.099869575	0.002344276	7.979895485	1.09E–08	7.22E–05	chr19	1074926
cg15944026	0.119332817	0.002164169	7.882586644	1.38E–08	9.07E–05	chr14	91883673
cg06451157	0.935929498	−0.001695081	−7.873892837	1.41E–08	9.14E–05	chr6	29868295
cg26965779	0.60414042	−0.00413718	−7.829559537	1.58E–08	0.000100155	chr6	29854089
cg08527435	0.875768342	−0.002675845	−7.82676586	1.59E–08	0.000100155	chr19	2200504
cg12998850	0.11986598	0.001545831	7.614691339	2.70E–08	0.000168189	chr6	29894946
cg11086883	0.557438738	−0.003588079	−7.596788883	2.83E–08	0.000173171	chr6	29908891
cg08661164	0.849490099	−0.002406106	−7.59305842	2.85E–08	0.000173171	chr1	93301669
cg04614203	0.563177139	−0.001781373	−7.57557512	2.98E–08	0.000178744	chrX	55114262
cg22167763	0.887249686	−0.002826962	−7.498662376	3.62E–08	0.000214463	chr13	95315772
cg10804687	0.955599083	−0.001487851	−7.463057535	3.96E–08	0.000231893	chr6	29859520
cg25650661	0.100941361	0.001609956	7.442407358	4.18E–08	0.000241465	chr6	29856278
cg22956635	0.975757794	−0.002854309	−7.380301859	4.89E–08	0.000279462	chr17	56566398
cg13946520	0.095467324	0.001748323	7.356583621	5.20E–08	0.000293426	chr2	87017689

cg14449180	0.109252657	0.002217793	7.326107969	5.62E–08	0.000313514	chr6	29894619
cg14766769	0.754409595	−0.002209626	−7.299549423	6.01E–08	0.00033172	chr9	125874000
cg17924072	0.115567047	0.001828031	7.294686825	6.09E–08	0.000332097	chr19	1075067
cg21836699	0.911261366	−0.001472	−7.240950969	6.99E–08	0.00037686	chr4	9557805
cg21810411	0.974657957	−0.007308597	−7.234503458	7.10E–08	0.000378933	chr6	32549139
cg06517984	0.879962938	−0.001196699	−7.204715381	7.67E–08	0.000404597	chr8	143407646
cg05088017	0.856357281	−0.002504125	−7.160234685	8.60E–08	0.000448742	chr15	22368957
cg01388630	0.968328655	−0.00733015	−7.112018414	9.73E–08	0.000502698	chr6	29817024
cg25023257	0.934770581	−0.001647131	−7.091748339	1.03E–07	0.000524118	chr1	203146223
cg02050694	0.125468346	0.002790783	7.058491327	1.12E–07	0.000565197	chr9	14346393
cg05834805	0.103843588	0.001380388	7.049792625	1.14E–07	0.000572097	chr9	123631524
cg20451455	0.875133306	−0.003198385	−7.029911954	1.20E–07	0.000596154	chr1	3449825
cg15697453	0.502760367	−0.001665995	−7.013280216	1.26E–07	0.000616108	chr6	31323985
cg15909951	0.092348646	0.000741771	6.96719478	1.42E–07	0.000687384	chr2	242743702
cg13470719	0.755037215	−0.002304518	−6.932595454	1.55E–07	0.000744591	chr7	157004224
cg07291958	0.081874288	0.001325477	6.922748247	1.59E–07	0.000756416	chr2	26785531
cg07731312	0.890214652	−0.000777394	−6.88507357	1.75E–07	0.000826261	chr16	88887321
cg14382215	0.81167243	−0.001961227	−6.805289827	2.16E–07	0.001007737	chr1	161169007
cg22989463	0.915630564	−0.001811943	−6.800705103	2.18E–07	0.001010175	chr8	12550605
cg03036047	0.946668321	−0.007087916	−6.783323934	2.29E–07	0.001047185	chr6	32362744
cg04214430	0.874415473	0.001036927	6.769622351	2.37E–07	0.001075275	chr13	50702707
cg04917511	0.044928388	0.002435103	6.740394673	2.56E–07	0.001143834	chr2	26785460
cg20465661	0.886556787	−0.002592404	−6.738958117	2.57E–07	0.001143834	chr1	248551409
cg05095290	0.205142571	0.002751738	6.709762552	2.77E–07	0.00121627	chr14	103995143
cg17431052	0.14457338	0.001655204	6.708615417	2.78E–07	0.00121627	chr19	37742481
cg03742947	0.105790242	0.000814475	6.654511787	3.21E–07	0.001389534	chr6	29856275
cg23619824	0.929118928	−0.000498618	−6.640960718	3.32E–07	0.001417162	chr10	133274140
cg10150686	0.046468125	0.002708313	6.637956414	3.35E–07	0.001417162	chr2	26785332
cg13667021	0.905294491	−0.002371802	−6.636982411	3.36E–07	0.001417162	chr11	124311423
cg04373948	0.784264643	−0.003027603	−6.564547663	4.06E–07	0.001697753	chr12	90806795
cg25043378	0.763283027	−0.002425439	−6.561905767	4.09E–07	0.001697753	chr8	41522857
cg14761252	0.09153213	0.001601376	6.542065019	4.31E–07	0.001773914	chr18	71815123
cg25668922	0.110539345	0.000678789	6.360939757	6.97E–07	0.002842935	chr5	34656126
cg23533270	0.80369607	−0.002843815	−6.317957981	7.81E–07	0.003160894	chr10	78635553
cg26133206	0.107519123	−0.000725855	−6.294124679	8.32E–07	0.003340285	chr5	133861746
cg05019905	0.116206299	0.002620094	6.281735207	8.60E–07	0.003424159	chr6	29894831
cg12477902	0.118986226	0.001341872	6.269535264	8.89E–07	0.003508649	chr19	44599083
cg00879843	0.084212113	0.000647233	6.241988783	9.57E–07	0.003745864	chr6	33239764
cg03091512	0.063351544	0.000911903	6.18182351	1.12E–06	0.004347943	chr2	227701050
cg14093630	0.149284409	−0.000961515	−6.180237109	1.13E–06	0.004347943	chr9	15423618
cg15547734	0.93231441	−0.000863484	−6.163797757	1.18E–06	0.004503819	chr5	45262443
cg07418114	0.785825968	−0.00160756	−6.161191731	1.19E–06	0.004503819	chr1	156836717
cg15931205	0.160724939	0.003341091	6.126381878	1.30E–06	0.004905636	chr6	29894820
cg16896847	0.060169672	0.001468019	6.050937448	1.60E–06	0.005960118	chr8	144512042
cg05299140	0.813821704	−0.003565115	−6.025143509	1.71E–06	0.006339127	chr17	47972401
cg05581880	0.927492565	−0.001341432	−6.017877232	1.74E–06	0.00641521	chr7	224681

cg04023150	0.044194338	0.001003149	6.014278858	1.76E–06	0.00642888	chr1	44873064
cg25623768	0.118836824	0.002357002	5.952503948	2.08E–06	0.007535102	chr2	45241008
cg16832267	0.130913979	0.002148988	5.908772827	2.34E–06	0.008414941	chr2	18060102
cg00599809	0.86203332	−0.001223808	−5.895589996	2.42E–06	0.008640461	chr7	72411550
cg23092777	0.060654055	0.000724424	5.893516802	2.44E–06	0.008640461	chr12	72057772
cg15631337	0.072270047	0.000629118	5.868250728	2.61E–06	0.009183192	chr17	46048070
cg14344315	0.08761901	0.000979463	5.838558648	2.83E–06	0.009878381	chr15	31733445
cg05049361	0.103341681	0.001306761	5.795058362	3.18E–06	0.011031733	chr14	69726536
cg06212289	0.138909453	0.001036915	5.78249591	3.29E–06	0.011264362	chr6	170606058
cg17583158	0.872685664	−0.003351996	−5.782100863	3.29E–06	0.011264362	chr8	2037874
cg25662463	0.092764752	0.001369246	5.778123722	3.33E–06	0.01130661	chr10	118897857
cg10151583	0.893772105	−0.001223373	−5.737542241	3.72E–06	0.012531927	chr16	88460502
cg09258813	0.11733558	0.002004469	5.72214111	3.88E–06	0.012975714	chr8	37823409
cg18875551	0.948530183	−0.000428601	−5.68355517	4.30E–06	0.014307665	chr1	24967164
cg24793642	0.747818058	−0.003370601	−5.674079668	4.41E–06	0.014580233	chr8	57351185
cg26230285	0.090621705	0.001164179	5.638193561	4.87E–06	0.015962711	chr15	26108412
cg02149708	0.114233734	0.000785708	5.601774517	5.37E–06	0.017503642	chr6	166582159
cg02737759	0.087075912	0.000506665	5.586412124	5.60E–06	0.01812794	chr12	72057786
cg04460984	0.13153616	0.001317538	5.578810956	5.72E–06	0.018383696	chr15	78933630
cg13617889	0.082900189	0.001396906	5.559412896	6.03E–06	0.019251451	chr15	31733101
cg25149122	0.737303898	−0.002494002	−5.509560714	6.90E–06	0.021902705	chr18	74201231
cg17636541	0.064957284	0.000544678	5.493951516	7.20E–06	0.022704689	chr6	30658493
cg03182504	0.082141817	−0.000401077	−5.490685815	7.27E–06	0.0227596	chr7	144532459
cg27589088	0.042376481	0.00158068	5.478647325	7.51E–06	0.023367002	chr16	80966033
cg19334406	0.942167223	−0.00053392	−5.473377616	7.62E–06	0.02355366	chr11	126319635
cg14455307	0.055059452	0.000398969	5.470302296	7.68E–06	0.023601369	chr18	60987429
cg17861154	0.162777958	0.000815029	5.464381808	7.81E–06	0.02383406	chr10	102509693
cg25226657	0.052330079	0.003594178	5.460833773	7.88E–06	0.023915032	chr5	149110983
cg02603304	0.904568683	−0.000810497	−5.457990582	7.94E–06	0.023951213	chr6	29865979
cg19633205	0.277998136	0.003030554	5.452785238	8.06E–06	0.024143196	chr1	35544470
cg13056744	0.070928104	0.0022588	5.429470453	8.58E–06	0.025568352	chr6	29894940
cg22488268	0.132174271	0.001239858	5.420770332	8.79E–06	0.025996228	chr7	1498641
cg02021485	0.886470099	−0.000885423	−5.418903167	8.83E–06	0.025996228	chr11	65959730
cg14190889	0.643665645	−0.004237184	−5.41245068	8.99E–06	0.026297966	chr6	29854174
cg00369443	0.907781694	−0.001861929	−5.398024981	9.35E–06	0.027188669	chr7	157918647
cg23130010	0.173066886	0.00234084	5.365656134	1.02E–05	0.029520193	chr6	29855462
cg27580026	0.079909745	0.001125994	5.349179782	1.07E–05	0.030694162	chr2	27717965
cg08934976	0.929530545	−0.000400919	−5.335793983	1.11E–05	0.031515393	chr5	1264529
cg20839522	0.93645383	−0.003638821	−5.334470419	1.11E–05	0.031515393	chr4	132649479
cg22819767	0.884815026	−0.001235815	−5.333048994	1.12E–05	0.031515393	chr10	11866910
cg01336390	0.259094174	0.002990316	5.323027992	1.15E–05	0.03208135	chr6	29895059
cg03339321	0.866078076	−0.00327321	−5.322287145	1.15E–05	0.03208135	chr6	30080642
cg06407657	0.876292765	−0.000716819	−5.318242698	1.16E–05	0.032251962	chr20	43937138
cg19707379	0.970318371	−0.000794704	−5.311092321	1.19E–05	0.032700518	chr17	78882693
cg27153492	0.121151376	0.002333435	5.304017771	1.21E–05	0.033149576	chr18	13801010

cg07237326	0.121431664	0.00227122	5.277255125	1.30E–05	0.035414151	chr19	37742739
cg26220528	0.05026994	0.00106936	5.275689039	1.31E–05	0.035414151	chr12	57623348
cg09335658	0.791837037	−0.00236892	−5.264847076	1.34E–05	0.036275319	chr7	127961612
cg18052778	0.857756017	−0.000541725	−5.257578	1.37E–05	0.036584781	chr17	46030312
cg27133432	0.920123594	0.00071672	5.257094357	1.37E–05	0.036584781	chr15	63026360
cg09086151	0.942467846	−0.001378821	−5.255681266	1.38E–05	0.036584781	chr6	32550067
cg16587443	0.189279625	−0.001189885	−5.24385661	1.42E–05	0.037376759	chr4	144434046
cg25130590	0.968246967	−0.007199049	−5.244236249	1.42E–05	0.037376759	chr18	77110161
cg01379290	0.763159542	−0.001147952	−5.206643285	1.58E–05	0.041152713	chr2	240679638
cg25797055	0.066262269	0.001401381	5.204047629	1.59E–05	0.041223893	chr4	141490097
cg12407979	0.631341352	−0.002121991	−5.18524671	1.67E–05	0.043165623	chr11	104817914
cg12013713	0.77064011	−0.00113032	−5.181236709	1.69E–05	0.043410213	chr7	139760671
cg01962869	0.109759133	0.000736063	5.165215334	1.77E–05	0.045113951	chr5	172482723
cg06181784	0.08049117	0.000707046	5.158086586	1.80E–05	0.045760491	chr3	9993934
cg18477943	0.9619185	−0.00179747	−5.151692423	1.83E–05	0.04632453	chr3	66002958
cg12008501	0.917225983	−0.000470675	−5.145012651	1.87E–05	0.046875485	chr2	208678058
cg25071520	0.161717442	0.000685464	5.143573721	1.87E–05	0.046875485	chr16	49910333
cg01499815	0.096169419	0.003215416	5.136612529	1.91E–05	0.047530766	chr6	29895074
cg26140688	0.155258019	0.001056304	5.12903875	1.95E–05	0.048277272	chr21	33245471
cg08534175	0.858182739	−0.001578904	−5.120460752	2.00E–05	0.049171599	chr16	2964815
cg24668883	0.854755145	−0.001226592	−5.117375996	2.01E–05	0.049222008	chr5	17257844
cg21279316	0.101472294	0.000723362	5.116390133	2.02E–05	0.049222008	chr15	64915229
cg22734086	0.131178675	0.002177675	5.101900288	2.10E–05	0.050954529	chr2	26785367
cg03638937	0.899692614	0.000344158	5.099149062	2.11E–05	0.051083749	chr11	393570
cg19117063	0.85992299	−0.002057472	−5.090451512	2.17E–05	0.052053837	chr6	32365553
cg27114028	0.900260914	−0.00046465	−5.08237003	2.21E–05	0.052954441	chr12	122217423
cg15654458	0.959023791	−0.006989363	−5.078898925	2.24E–05	0.053197258	chr6	88053106
cg20673481	0.090080495	0.000767717	5.073037876	2.27E–05	0.053792791	chr2	18059454
cg21463869	0.934615773	−0.000464212	−5.061301089	2.35E–05	0.055277157	chr8	39442078
cg08022819	0.824545402	−0.000528015	−5.052812137	2.40E–05	0.056301739	chr16	29884806
cg17966192	0.203473908	0.003420789	5.037511209	2.50E–05	0.058424749	chr2	108994116
cg00010108	0.882981709	−0.000641968	−5.025215184	2.59E–05	0.060133233	chr4	1615271
cg18349863	0.479330229	−0.002604841	−5.016824884	2.65E–05	0.061037222	chr6	29912713
cg12602374	0.09471996	0.000452137	5.015208581	2.66E–05	0.061037222	chr5	38557162
cg14601050	0.960149108	−0.000767064	−5.014545542	2.67E–05	0.061037222	chr17	73727533
cg25302936	0.092171499	0.000678438	5.011189081	2.69E–05	0.061310766	chr8	67579596
cg09428031	0.07654756	0.000744376	5.007209179	2.72E–05	0.061691973	chr4	48782361
cg11432034	0.082187228	0.000368601	4.997772224	2.79E–05	0.062822668	chr3	13520913
cg12944659	0.778370494	−0.006796246	−4.997164813	2.79E–05	0.062822668	chr11	15957513
cg00916536	0.1807976	0.001488341	4.994288474	2.82E–05	0.063026919	chr2	87017419
cg09510924	0.874339241	0.000757512	4.987456654	2.87E–05	0.063794033	chr17	17860297
cg11752440	0.081788321	0.00058898	4.986508939	2.88E–05	0.063794033	chr11	102980466
cg05600935	0.808381342	−0.000655049	−4.982136889	2.91E–05	0.064211843	chr7	102132012
cg19242610	0.933503472	−0.003335792	−4.980796883	2.92E–05	0.064211843	chr1	161569954
cg12341757	0.128318451	0.000591978	4.97855039	2.94E–05	0.064316472	chr8	134582241
cg09059250	0.948554195	−0.000756917	−4.974237475	2.98E–05	0.064787627	chr12	98095166
cg15671450	0.111227399	0.006058617	4.970621137	3.01E–05	0.065139355	chr6	29895116

cg26967186	0.929026631	−0.001202011	−4.963801553	3.06E–05	0.066070486	chr7	157919649
cg18049513	0.945494592	−0.000444641	−4.948381637	3.19E–05	0.068610944	chr11	541660
cg14403130	0.092025438	0.000703841	4.927188185	3.38E–05	0.072384228	chr1	44873057
cg06586505	0.919720343	−0.000356082	−4.91839684	3.47E–05	0.073820135	chr13	112611028
cg19540702	0.070819942	0.000912555	4.912328107	3.52E–05	0.074476063	chr19	19281175
cg04931090	0.157107743	0.000637595	4.911967513	3.53E–05	0.074476063	chr6	35181888
cg03156904	0.114312473	0.002717599	4.905474536	3.59E–05	0.075481919	chr18	13801143
cg06345693	0.920127933	−0.00044612	−4.893449607	3.71E–05	0.077335385	chr12	133158310
cg14951598	0.388958365	−0.000993076	−4.893846407	3.71E–05	0.077335385	chr6	41472931
cg16776350	0.136285511	0.0009883	4.891740629	3.73E–05	0.077365555	chr1	160549158
cg16487237	0.093760386	0.000631253	4.883922377	3.81E–05	0.078269087	chr3	137487248
cg19458602	0.922234544	−0.000514982	−4.884263076	3.81E–05	0.078269087	chr19	7992003
cg20767025	0.091233436	0.000520609	4.882645098	3.82E–05	0.078269087	chr7	128431850
cg24797456	0.954984332	−0.001441798	−4.881294824	3.84E–05	0.078269087	chr4	76622044
cg00952054	0.881183598	−0.00056435	−4.858797001	4.08E–05	0.082436561	chr1	9437047
cg19835136	0.869850851	−0.000897008	−4.858935453	4.08E–05	0.082436561	chr19	17650995
cg07053873	0.111403503	−0.00070201	−4.855244723	4.12E–05	0.082436561	chr11	117049953
cg01962146	0.226519828	0.001066526	4.854220747	4.13E–05	0.082436561	chr4	995931
cg09948336	0.867760011	0.000883983	4.85477096	4.13E–05	0.082436561	chr11	64394492
cg24509591	0.94278851	0.000308972	4.853213804	4.14E–05	0.082436561	chr8	75779095
cg22488797	0.142373594	0.002152587	4.85061986	4.17E–05	0.082684366	chr5	134363324
cg20313496	0.162942352	0.001492582	4.847299362	4.21E–05	0.083099186	chr1	155579807
cg07274406	0.063027355	0.000581754	4.845571718	4.23E–05	0.083154568	chr21	46334192
cg14258143	0.120848596	0.00111668	4.829399822	4.42E–05	0.086562772	chr1	32714146
cg15925143	0.831760493	−0.00131757	−4.826454624	4.46E–05	0.086912044	chr6	170575847
cg25643819	0.188115741	0.001382865	4.82330272	4.50E–05	0.087313444	chr6	30227942
cg20313856	0.871137817	−0.001221699	−4.820395623	4.53E–05	0.08765941	chr2	38825345
cg21412456	0.834894785	−0.004485084	−4.782742688	5.02E–05	0.096775651	chr1	26608782
cg02779467	0.088165314	0.001184856	4.780692013	5.05E–05	0.096868598	chr17	18761289
cg04682862	0.126435355	0.001018565	4.779498763	5.07E–05	0.096868598	chr16	66304367
cg10642820	0.074310271	0.000373523	4.767088319	5.24E–05	0.099817164	chr5	64777786
cg01747792	0.101937908	0.000768892	4.764461476	5.28E–05	0.100143509	chr20	61806628
cg01952509	0.115690495	−0.000407107	−4.746180314	5.55E–05	0.104863131	chr3	44380113
cg25852545	0.075922817	−0.000301738	−4.732972271	5.75E–05	0.108294768	chr3	113775465
cg09746279	0.130911001	0.000823382	4.721949429	5.93E–05	0.111174345	chr6	29895016
cg24878755	0.058516668	−0.000391051	−4.71919711	5.98E–05	0.111582494	chr6	139349590
cg18801028	0.903454526	−0.000471008	−4.715708993	6.03E–05	0.111926797	chr1	29646475
cg22766014	0.05337914	0.000304126	4.71526378	6.04E–05	0.111926797	chr2	69241106
cg02836479	0.040330568	0.00033023	4.713533956	6.07E–05	0.11202929	chr16	21964489
cg23099265	0.905457089	−0.003740647	−4.706431021	6.19E–05	0.113244587	chr7	1515564
cg02927058	0.922639635	−0.000636483	−4.705430797	6.20E–05	0.113244587	chr1	10116780
cg22677240	0.678201562	−0.001799194	−4.705732524	6.20E–05	0.113244587	chr1	147802979
cg26853340	0.897010561	−0.000860147	−4.699480724	6.31E–05	0.114668775	chr15	81409649
cg16676472	0.33224467	−0.002245031	−4.689106344	6.49E–05	0.117523745	chr19	42363949
cg12306213	0.087258851	0.000440607	4.67810379	6.69E–05	0.120657878	chr6	71666824
cg17073392	0.116980145	0.002042516	4.65839184	7.05E–05	0.126857454	chr15	69324108
cg24426536	0.118999282	0.001190257	4.656969862	7.08E–05	0.126880901	chr1	161500610

cg04284317	0.114555765	−0.000474081	−4.653897372	7.14E–05	0.127479119	chr10	120101832
cg08057411	0.899850756	−0.000525032	−4.650392998	7.21E–05	0.128232927	chr16	84988003
cg03045133	0.879293342	−0.003121566	−4.645063598	7.32E–05	0.129165257	chr6	29870056
cg12733040	0.921294971	−0.000631431	−4.644152434	7.33E–05	0.129165257	chr22	17303493
cg21190253	0.927538209	−0.000659362	−4.643734682	7.34E–05	0.129165257	chrX	33231153
cg07578215	0.928053416	−0.000475047	−4.635190711	7.52E–05	0.131735212	chr1	236157114
cg15975217	0.095187527	0.001135726	4.633628402	7.55E–05	0.131822177	chr4	140656926
cg14866419	0.090010841	−0.000575617	−4.622602237	7.78E–05	0.135166264	chr14	100704911
cg04310824	0.075999594	0.000394323	4.621540098	7.80E–05	0.135166264	chr1	53393297
cg09464883	0.946348167	−0.000411118	−4.620434895	7.82E–05	0.135166264	chr4	79689621
cg07873178	0.104193922	0.000547552	4.619212201	7.85E–05	0.135166264	chr11	65307616
cg13457172	0.133325677	0.001054331	4.617718444	7.88E–05	0.135238704	chr10	118897847
cg10890016	0.925603846	0.000609194	4.608151343	8.09E–05	0.138326283	chr7	885027
cg03292213	0.108769443	0.00316018	4.60657035	8.13E–05	0.138436716	chr17	17109640
cg02880176	0.128238172	0.001577418	4.600466452	8.26E–05	0.140268903	chr17	17109817
cg24433586	0.891860697	−0.000668306	−4.592222665	8.45E–05	0.142958821	chr17	79210494
cg27544294	0.67499761	−0.001095846	−4.589789659	8.51E–05	0.143410924	chr22	25082493
cg11050622	0.072360537	0.000823623	4.585319606	8.61E–05	0.14466762	chr14	31090974
cg18805978	0.914160798	−0.000634198	−4.579000604	8.76E–05	0.146674626	chr2	130734711
cg23003881	0.054853025	0.000612604	4.570684366	8.96E–05	0.149320708	chr5	173043929
cg02110963	0.929078123	−0.000547945	−4.569922797	8.98E–05	0.149320708	chr6	10434975
cg11802806	0.909639683	−0.000368488	−4.568002487	9.03E–05	0.149592253	chr1	25748133
cg14141549	0.89643638	−0.001605693	−4.552226914	9.42E–05	0.155634754	chr7	157919408
cg11149073	0.076591289	−0.00038044	−4.545795405	9.59E–05	0.157796756	chr17	11900221
cg17428043	0.056233648	0.000678751	4.544677867	9.62E–05	0.157796756	chr1	180200104
cg12304937	0.539549857	0.001900768	4.53929317	9.76E–05	0.159036393	chr7	680013
cg04259358	0.07346013	0.000650348	4.538106288	9.79E–05	0.159036393	chr10	119590450
cg14099398	0.186308635	0.002505866	4.538341238	9.79E–05	0.159036393	chr17	17109800
cg12877335	0.46190966	0.002739542	4.53562813	9.86E–05	0.159344229	chr12	94539319
cg09678228	0.115043022	−0.000780213	−4.534950212	9.88E–05	0.159344229	chr12	44230058
cg05037927	0.121111067	0.001604273	4.520929443	0.000102635	0.165001025	chr2	61372117
cg11290181	0.850960414	−0.002666254	−4.515323023	0.000104214	0.166503225	chr5	130604045
cg11528572	0.779837022	0.00077958	4.515179559	0.000104255	0.166503225	chr7	6915508
cg24538947	0.124431377	0.000882256	4.511152303	0.000105405	0.167754161	chr2	26785301
cg18697487	0.928399176	−0.000518929	−4.51002435	0.000105729	0.167754161	chr19	1502095
cg22338567	0.095321338	0.00088163	4.507773895	0.000106379	0.167780927	chr7	107642444
cg05338731	0.029586107	0.005152326	4.507573942	0.000106437	0.167780927	chr22	23489041
cg23543615	0.064419577	0.001312361	4.500790452	0.000108422	0.170356344	chr19	19281185
cg10005475	0.885424623	−0.000891348	−4.495904675	0.000109874	0.171359058	chr17	74011063
cg09453870	0.112789648	0.001777457	4.493022275	0.00011074	0.171359058	chr15	69324156
cg09199225	0.954975346	−0.004142846	−4.492989646	0.00011075	0.171359058	chr6	32149260
cg04927341	0.95101204	−0.000642985	−4.492871565	0.000110785	0.171359058	chr1	86576497
cg22502715	0.127873201	0.002683032	4.492740681	0.000110825	0.171359058	chr1	247171572
cg06902219	0.123487162	0.002525693	4.490979245	0.000111358	0.171636282	chr6	29856294
cg26092192	0.933036226	−0.000930873	−4.487578157	0.000112394	0.172684851	chr17	80759678
cg15549251	0.945749217	−0.000506464	−4.482617688	0.000113922	0.17448061	chr15	40426006
cg05442111	0.660676876	0.000853368	4.476634532	0.000115792	0.176053785	chr11	89901285

cg17653969	0.943212799	−0.000509176	−4.47537295	0.000116191	0.176053785	chr1	229439834
cg23070111	0.100166411	0.000423679	4.474766636	0.000116383	0.176053785	chr1	186649985
cg18881269	0.888510759	−0.000458198	−4.474713959	0.000116399	0.176053785	chr12	6936267
cg07139509	0.178658928	0.002907067	4.469490701	0.000118066	0.177794759	chr14	70038717
cg26473478	0.104468748	0.002296888	4.468816566	0.000118283	0.177794759	chr6	30614823
cg04724720	0.124701505	−0.00044323	−4.462351737	0.000120382	0.180157091	chr11	65640939
cg01667185	0.886408539	−0.000559745	−4.461698201	0.000120597	0.180157091	chr11	66625638
cg08362486	0.933685473	−0.000318986	−4.458495879	0.000121652	0.18063424	chr16	4935615
cg02230705	0.082523635	0.000625103	4.458471628	0.00012166	0.18063424	chr4	100867350
cg00963654	0.118143932	0.00221996	4.454930837	0.000122838	0.181826901	chr18	13801271
cg05948856	0.886753418	−0.000443575	−4.450997103	0.00012416	0.182954999	chr19	3982825
cg00634652	0.896783366	0.000750265	4.449556011	0.000124647	0.182954999	chr13	112166963
cg04186471	0.92017841	−0.000326629	−4.449311034	0.00012473	0.182954999	chrX	153523440
cg22161299	0.547767139	0.000863014	4.44670974	0.000125616	0.183400701	chr19	11468272
cg11224271	0.86359668	−0.000507794	−4.446202263	0.00012579	0.183400701	chr3	129744515
cg18957138	0.075720266	0.000271954	4.437719301	0.000128726	0.187119901	chr9	73027331
cg17508462	0.097863506	0.000494422	4.432517825	0.00013056	0.189219296	chr5	180542340
cg20971958	0.906547361	−0.001500561	−4.424342336	0.000133495	0.192897286	chr4	1594894
cg26463106	0.104151195	0.001105056	4.421196096	0.000134642	0.193977208	chr3	9404837
cg12030638	0.620803557	0.0010577	4.419246981	0.000135357	0.194430938	chrX	133675340
cg26801241	0.16901549	0.00090033	4.416163196	0.000136497	0.195489494	chr16	47495768
cg17988326	0.783915748	0.001044757	4.413010539	0.000137672	0.196429069	chr1	41091703
cg05704893	0.077568024	0.000285431	4.412235567	0.000137962	0.196429069	chr17	7982773
cg17163751	0.102910303	−0.000473405	−4.407312383	0.000139821	0.198493289	chr10	71812596
cg04864199	0.07551187	−0.000423976	−4.405086819	0.000140669	0.199115408	chr15	45493327
cg03958058	0.969342626	−0.007193203	−4.403470567	0.000141288	0.19941062	chr10	133761284

Supplementary Table S4: CpGs associated with progesterone levels in PCOS patients

Row.names	intercept	beta	t	pval	qval	chr	pos
cg00762003	1.098857134	−0.616724321	−6.64908018	3.25E−07	0.056684002	chr21	45393541
cg08188400	0.126165778	0.046313032	6.546351782	4.26E−07	0.056684002	chr19	7969347
cg07286123	0.102539658	0.035158316	6.542625459	4.30E−07	0.056684002	chr20	23067126
cg09546220	0.86888161	−0.050653241	−6.280399517	8.63E−07	0.085272869	chr21	40036490
cg02496295	0.171304643	0.07600485	6.19650408	1.08E−06	0.085348933	chr4	2846155
cg20947082	0.386072142	0.05318017	6.085557348	1.45E−06	0.095739393	chr1	233749643
cg15361028	0.106809472	0.033900248	6.00556114	1.80E−06	0.101737571	chr6	53658683
cg26847156	0.947833861	−0.060621147	−5.775799583	3.35E−06	0.136502163	chr18	10550303
cg07455790	0.86261056	−0.139314841	−5.773635372	3.37E−06	0.136502163	chr6	31650735
cg18726518	0.958558694	−0.028964356	−5.756047821	3.54E−06	0.136502163	chr12	132924516
cg03580106	0.156620317	0.104059743	5.684403084	4.29E−06	0.136502163	chr16	89283057
cg26775843	0.143536217	0.066163413	5.681400005	4.33E−06	0.136502163	chr4	88343678
cg22211395	0.028264357	0.043649311	5.667635755	4.49E−06	0.136502163	chr5	80597321
cg00738945	0.834725938	0.064204995	5.610385075	5.25E−06	0.140826711	chr6	32340354
cg00173538	0.831999484	0.047928257	5.602336074	5.36E−06	0.140826711	chr6	42071219
cg03190929	0.937169066	−0.026025455	−5.557803459	6.05E−06	0.140826711	chr17	38075855
cg21298523	−0.036778528	0.36120398	5.532864798	6.48E−06	0.140826711	chr4	88343936
cg13940125	0.999138128	−0.219025372	−5.51228039	6.85E−06	0.140826711	chr1	197237119

cg06128688	0.395258122	−0.126079643	−5.49526667	7.18E–06	0.140826711	chr12	96883733
cg09817985	0.75962029	0.098774591	5.491054892	7.26E–06	0.140826711	chr20	25054507
cg23522832	0.087983364	0.021278099	5.479690659	7.49E–06	0.140826711	chr1	45769727
cg24856518	0.08016155	0.024899728	5.424092067	8.71E–06	0.15640179	chr2	120517676
cg08428486	0.898561846	−0.090272945	−5.369345666	1.01E–05	0.172930917	chr7	140452281
cg12905273	0.067532901	0.043649968	5.346996044	1.07E–05	0.172930917	chr6	33359440
cg10572624	0.859286085	−0.037291684	−5.340349521	1.09E–05	0.172930917	chr1	40428318
cg00484206	0.886855097	0.038066988	5.286763813	1.27E–05	0.186982636	chr16	66041033
cg13588800	0.358803292	−0.109242946	−5.266638145	1.34E–05	0.186982636	chr7	19155785
cg01546814	1.113419379	−0.5147582	−5.252685326	1.39E–05	0.186982636	chr16	4295972
cg19919446	0.107107906	0.033241105	5.242720891	1.43E–05	0.186982636	chr3	98241922
cg03774288	0.886771586	0.043049401	5.240846733	1.44E–05	0.186982636	chr10	131647543
cg24665612	1.111509811	−0.471811288	−5.232912236	1.47E–05	0.186982636	chrX	134971730
cg19796532	0.530991201	−0.138842486	−5.210535573	1.56E–05	0.191042237	chr12	50426901
cg07013326	0.197362117	0.08674269	5.202157316	1.60E–05	0.191042237	chr6	57037090
cg18264486	0.814866139	−0.222866994	−5.167952656	1.75E–05	0.197791888	chr6	31650916
cg10499042	0.943534533	−0.027587165	−5.163175652	1.78E–05	0.197791888	chr2	141650127
cg01904243	0.330719707	−0.103568522	−5.157600618	1.80E–05	0.197791888	chr14	74251254
